# Detecting the metabolic transition to personalize nutritional timing: model development and preliminary validation in a large ICU cohort

**DOI:** 10.1186/s13054-026-05874-5

**Published:** 2026-02-24

**Authors:** Yonatan Gargi, Neriya Levran, Jacob Vine, Amir Cohen, Dana Weiner, Dorit Stein, Ori Levi, Dor Cohen, Julia Klein, Hamutal S. Taube, Maxim Glebov, Teddy Lazebnik, Mor Saban, Shaked Efrat, Elad Drori, Yael Haviv, Eran Segal

**Affiliations:** 1 Departments of Anesthesiology and Intensive Care, Sheba Medical Center, Ramat Gan, Israel; 2 Department of Nutrition, Sheba Medical Center, Ramat Gan, Israel; 3 Department of Intensive Care, Sheba Medical Center, Ramat Gan, Israel; 4 Departments of Nutrition and Intensive Care, Sheba Medical Center, Ramat Gan, Israel; 5 Intensive Care Unit, Sheba Medical Center, Ramat Gan, Israel; 6 Universidad Favaloro, Buenos Aires, Argentina; 7 Department of Anesthesiology, Sheba Medical Center, Ramat Gan, Israel; 8 Department of Information Systems, University of Haifa, Haifa, Israel; 9 Department of Computing, Jonkoping University, Jonkoping, Sweden; 10 Gertner Institute for Epidemiology and Healthcare Research, Gray Faculty of Medical & Health Sciences, Tel Aviv University, Tel Aviv, Israel; 11 Department of Clinical Pharmacology, Sheba Medical Center, Ramat Gan, Israel; 12 Department of Intensive Care, Maaynei Hayeshua, Bnei Brak, Israel

**Keywords:** Critical illness, Metabolism, Catabolism, Nutrition, Intensive care, Insulin resistance

## Abstract

**Background:**

The metabolic transition from catabolism to anabolism is a key determinant of recovery in critical illness and should guide nutritional therapy. However, no validated clinical marker currently exists to identify this transition, and current clinical practice relies on calendar-based recommendations. We aim to develop and preliminarily validate a physiology-based, trajectory-driven model to detect the metabolic transition window in critically ill patients.

**Methods:**

We conducted a retrospective cohort study in a tertiary-care general ICU. A daily insulin resistance index (IRI) was computed from glucose and insulin data and corrected for steroid exposure. Transition was defined as a ≥ 30% sustained drop in IRI after its peak, together with ≥ 2 of 8 physiologic recovery criteria (lactate, noradrenaline, vasopressin, adrenaline, inflammatory markers-WBC, %neutrophils, CRP, and albumin). Associations with 90-day mortality and caloric exposure were evaluated using Kaplan–Meier analysis and multivariable landmark Cox models.

**Results:**

The cohort included 2,350 patients (age 59.8 ± 16.5 years; SOFA 11.7 ± 3.9; 82% mechanically ventilated). A metabolic transition was identified in 94% of patients, with ~ 60% transitioning by ICU day 3. Transition by day 3 was associated with lower 90-day mortality (HR 0.72, 95% CI 0.65–0.81). Patients who never transitioned had substantially higher mortality. High caloric delivery (≥ 1.0 kcal/kg/h for ≥ 24 h) before transition was independently associated with increased 90-day mortality (OR 1.25, 95% CI 1.01–1.55), with a dose–response pattern. In contrast, high caloric delivery based on calendar timing failed to demonstrate a similar pattern.

**Conclusions:**

We developed and validated a physiological model for detecting metabolic transition in critical illness and showed that transition occurs early and strongly predicts survival. Higher caloric delivery before transition is associated with increased mortality, supporting nutrition strategies aligned with physiological recovery rather than fixed calendar days.

**Supplementary Information:**

The online version contains supplementary material available at 10.1186/s13054-026-05874-5.

## Introduction

Optimal timing of nutritional support is an important but unresolved aspect of critical care. Critically ill patients typically traverse distinct metabolic phases. Initially, there is an intense catabolic “ebb” phase (first 24–48 h) characterized by tissue hypoperfusion and decreased overall metabolism, followed by a hypercatabolic “flow” phase (up to 7 days) during which significant protein degradation occurs [1, 2]. Subsequently, some patients enter a recovery/anabolic phase, while others may experience prolonged critical illness.

Both the European Society for Clinical Nutrition and Metabolism (ESPEN) and American Society for Parenteral and Enteral Nutrition (ASPEN) guidelines acknowledge these metabolic shifts and recommend strategies such as permissive underfeeding during the acute catabolic phase, with progressive advancement toward energy and protein targets by days 3–7 after ICU admission [3, 4]. However, both societies note that these recommendations are based on expert consensus and not on validated, physiology-driven criteria [3, 4].

A significant challenge to optimal nutritional provision remains- there is no single, validated clinical marker that precisely identifies the transition point from catabolism to anabolism. As a result, the optimal timing for reaching full nutritional targets is highly individualized and relies on multifactorial clinical assessment rather than a definitive “switch” [2–6].

Correctly identifying the timing of this transition is clinically crucial. Aggressive nutritional support during ongoing catabolism may worsen metabolic complications (hyperglycemia, refeeding syndrome, increased ureagenesis suggesting futile catabolism of provided proteins, telomere shortening and altered DNA methylation) and infectious complications as well as blunt beneficial autophagy and ketogenesis [7–13]. Recent large trials further suggest that higher dose or early administration of calories or protein may not improve outcomes and could be harmful in selected populations [7, 14–21]. On the other hand, delayed nutritional support after the onset of anabolism may impede recovery, muscle-mass restoration, and immune function [3–6, 13, 22].

Multiple surrogate approaches- nitrogen balance, C-reactive protein (CRP) kinetics, glucose-insulin dynamics, indirect calorimetry, as well as critical care metabolomics- have been proposed to characterize the metabolic shift [5, 23–26]. These are often constrained by technical complexity, intermittent sampling, or limited specificity, and are not routinely implemented at the bedside. Consequently, major trials and guidelines continue to rely on calendar-based criteria, rather than patient-specific-physiology, when determining nutritional targets [3, 4, 17–20, 27–29].

At the cellular level, sepsis impairs GLUT4 (Glucose Transporter Type 4) transcription and translocation, producing marked peripheral insulin resistance [30, 31]. Under these conditions, carbohydrate delivery predominantly raises circulating glucose rather than supporting muscle metabolism. These profound alterations in insulin sensitivity highlight the importance of metabolic trajectories during critical illness and suggest that dynamic glucose–insulin relationships may provide insight into the transition from catabolism to anabolism.

Observational and interventional studies highlight substantial inter-patient variability in the duration of catabolism and the optimal window for full nutritional support [7, 9, 14–19, 22, 27, 32–35], suggesting that the benefit of nutritional escalation may depend on *when* and *in whom* it is delivered. Emerging proposals and expert consensus therefore advocate for multi-parameter, trajectory-based definitions that incorporate dynamic changes in metabolic, inflammatory, and hemodynamic markers [25, 35, 36]. However, such models have not yet been systematically implemented or validated in large, real-world ICU cohorts.

To address this gap, we developed and tested a reproducible, trajectory-based model to detect the catabolic-to-anabolic transition in a large ICU cohort. The model integrates insulin resistance dynamics with hemodynamic, inflammatory, and metabolic parameters, applying predefined criteria for transition. We hypothesized that:

1. Earlier transition, as identified by this model, would be associated with improved 90-day mortality,
2. The provision of full caloric delivery before transition would be associated with increased mortality.

This framework may provide a physiology-based foundation for individualized, physiology guided nutrition strategies in critical care.

## Methods

### Study design and population

**Fig. 1:**
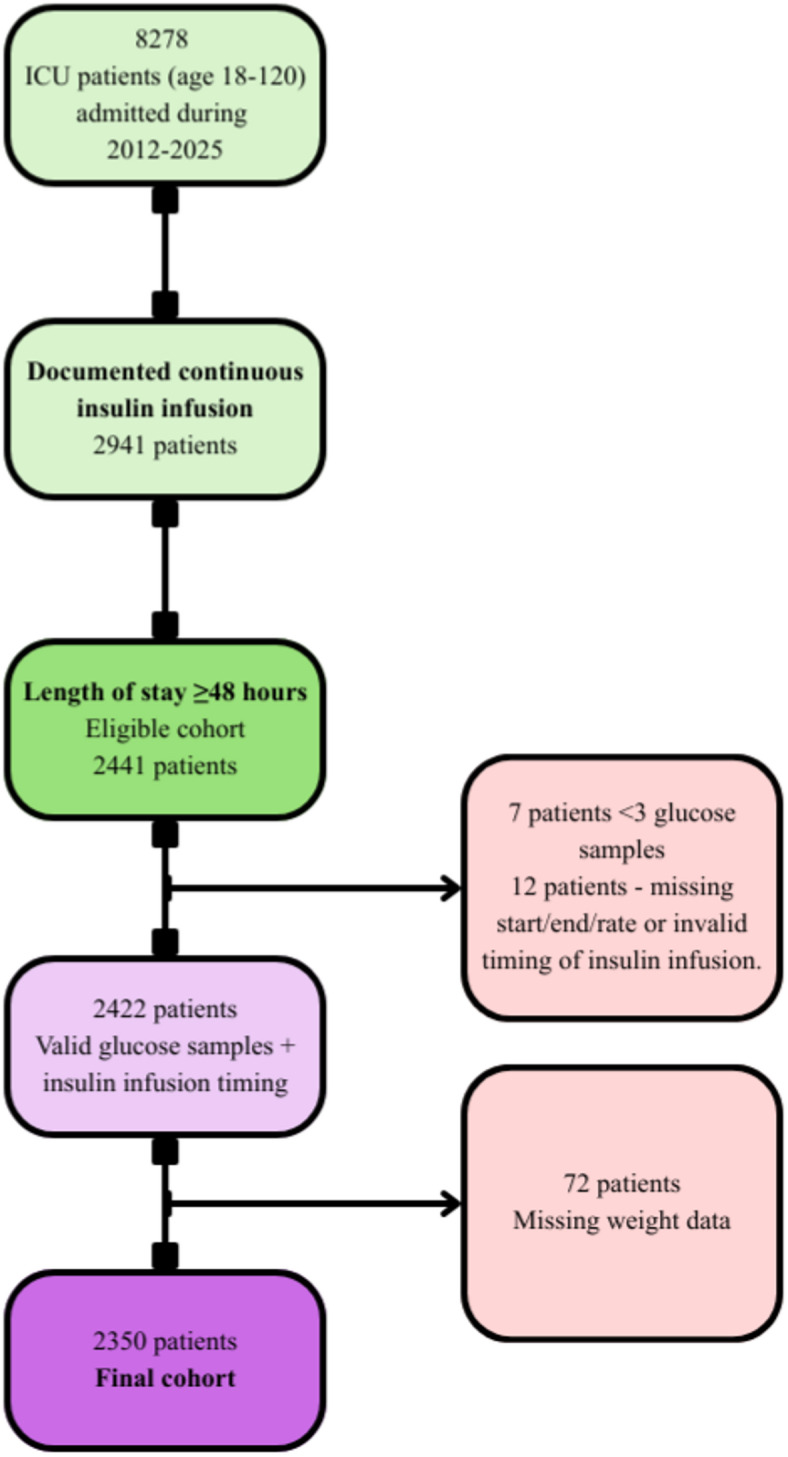
Cohort Selection Process for Final Study Population

We conducted a retrospective cohort study in the general ICU at Sheba Medical Center between January 2012 and Feb. 2025. Inclusion criteria were defined a priori and consisted of adult age (18–120 years) and an ICU length of stay ≥ 48 h. Analyses were restricted to admissions with extractable insulin infusion records, as insulin infusion time-series data are required to compute the insulin resistance index. We then applied pre-specified technical exclusions for inability to apply the transition model: fewer than three glucose measurements during the ICU stay, no valid continuous insulin infusion segment data (e.g., missing start/end time or rate, or invalid timing preventing IRI computation), or missing recorded body weight. The final study cohort is shown in Fig. 1.

### Definition of catabolic-to-anabolic transition

The primary variable of interest was the physiologic shift from catabolism to anabolism. We established an operational definition using a multi-parameter, trajectory-based model that combined insulin resistance dynamics with complementary metabolic and inflammatory markers.

Insulin resistance was quantified by an Insulin Resistance Index (IRI), calculated as:

$$\begin{aligned} {\text {IRI}} & =\left[ {\text {glucose} \times \left( {\mathrm{insulin}\,+\,0.5} \right)} \right] \times {\text {steroid correction factor}}\end{aligned}$$

with daily corticosteroid exposure incorporated as a hydrocortisone-equivalent adjustment. The steroid correction factors of 0.6, 0.8, 0.85 (based on hydrocortisone-equivalent dosing) were selected based on known effects of glucocorticoids on insulin resistance [37, 38] as well as extensive sensitivity and feature importance analysis (supplement 1 and 5). To avoid abrupt collapse of the IRI when insulin infusion was stopped, and to simulate a minimal basal endogenous insulin secretion [39], we added a constant of 0.5 U/h to the insulin infusion rate in the formula. Because this adjustment is a modeling assumption, we performed sensitivity analyses with alternative constants (0.1 and 1.0 U/h) and confirmed that transition rates, timing, and the association with mortality were robust to the exact value chosen.

A transition event was defined by a ≥ 30% decrease from the peak IRI, sustained below 70% of the peak for at least 24 h, allowing for brief (< 10%) rebounds. In addition, fulfillment of at least 2 of 8 physiologic criteria was required to establish transition: [1] a 30% decrease or normalization of lactate; [2] 30% reduction or cessation of norepinephrine [3], 30% reduction or cessation of adrenaline [4], 30% reduction or cessation of vasopressin; [5] a 40% decline or normalization of white blood cell count [6] a 30% decline or normalization of neutrophil percentage; [7] a 30% decrease in C-reactive protein (CRP); and [8] stabilization or increase in serum albumin. These parameters were selected a priori to capture complementary domains of recovery: (i) hemodynamic stabilization (vasopressor requirements, lactate clearance), (ii) resolution of systemic inflammation (WBC, neutrophils, CRP), (iii) attenuation of the stress response (rise or stabilization of albumin, as a negative acute-phase protein).

The selection of IRI reduction threshold, in addition to the number and thresholds of physiologic parameters, as well as use of nutrition at the denominator and the extent of steroid correction factor were informed by extensive sensitivity analyses, which demonstrated optimal outcome association strength and clinical feasibility across multiple tested configurations (Supplement 1).

### Study outcomes

The primary outcome was 90-day all-cause mortality, examined in relation to the timing of the catabolic-to-anabolic transition, which served as the main physiological exposure.

Secondary outcomes included exploratory analyses of calories delivery and overfeeding, and evaluation of the model’s robustness across heterogeneous clinical profiles.

Descriptive analysis included baseline characteristics, including comorbidities, and admission SOFA score.

### Data collection

Clinical and laboratory data were obtained from a comprehensive ICU database that includes serial measurements of glucose and insulin, calories intake, vasopressor administration, inflammatory markers, serum albumin, comorbid conditions, chronic medication use, ICU length of stay, and mortality.

### Data management and validation

For each patient, a dedicated audit log tracked data completeness, transition status, and trajectories of all included physiologic parameters, including exclusion reasons and key processing steps, to ensure reproducibility and model transparency. Delivered energy was quantified as hourly total kcal/kg/h.

### Data analysis

Baseline characteristics are presented as mean ± standard deviation (SD) for continuous variables, and as counts with percentages for categorical variables.

Model robustness was examined through sensitivity analyses varying IRI thresholds, steroid correction factors, and the number of required criteria. Subgroup analyses across major clinical phenotypes (e.g., sepsis, trauma, pancreatitis) provided internal validation, and an audit log was maintained for each patient to ensure reproducibility and transparency.

To further assess the potential causal relationship between the catabolic-to-anabolic transition and clinical outcomes, causal inference analyses were conducted using doubly robust estimation methods. Propensity scores for transition were derived from logistic regression models including demographic, clinical, and biochemical covariates measured prior to the transition. These scores were incorporated into inverse probability of treatment weighting (IPTW) and augmented inverse probability weighting (AIPW) frameworks to estimate the average treatment effect (ATE) on 90-day mortality and secondary recovery endpoints. Covariate balance before and after weighting was evaluated using standardized mean differences, and sensitivity analyses explored the robustness of causal estimates to unmeasured confounding using E-values and Rosenbaum bounds.

The primary outcome, 90-day all-cause mortality, was analyzed with Kaplan–Meier survival curves stratified by transition status at predefined landmark days ([3, 5, 7], and [10]), with differences assessed by the log-rank test. Landmark Cox proportional hazards models were constructed at each time point to estimate hazard ratios (HRs) with 95% confidence intervals (CIs) for the association between transition status and mortality. Multivariable models were adjusted for age, sex, baseline SOFA score, admission diagnoses, and major comorbidities. To mitigate immortal-time bias, patients who died or were censored before each landmark were excluded from the respective analysis. Changes in caloric intake before and after transition were assessed with paired and sign tests. A two-sided p value < 0.05 was considered statistically significant. Analyses were performed using Python (version 3.13.3).

### Sample size and precision

This retrospective cohort included all eligible ICU admissions; no a priori sample-size calculation was performed. We report the number at risk and the number of 90-day deaths at each landmark. At the day-3 landmark, 2,242 patients remained at risk and 848 deaths occurred within 90 days; with ~ 60% transitioned by day 3, the available number of events indicates adequate statistical information to detect moderate associations (approximately HR ≤ 0.82 or ≥ 1.22 at two-sided α = 0.05; Schoenfeld approximation).

## Results

The cohort included 2,350 ICU patients. Baseline demographics, admission diagnoses and comorbidities are detailed in Table 1. Admission diagnoses were mapped to APACHE III categories and recorded as co-existing conditions rather than a single primary diagnosis.

**Table 1:** Baseline demographic, clinical, and comorbidity characteristics of the study cohort

Characteristic	Overall (*n* = 2350)
Age, years	59.8 ± 16.5
SOFA score	11.7 ± 3.9
Length of stay	12.6 ± 13.0
Sex, n (%)	Male 1431 (60.9%)
**Admission diagnoses, n (%)**	
Sepsis (non-shock)	1152 (49.0%)
Septic shock	952 (40.5%)
Pneumonia	764 (32.5%)
Acute respiratory failure (non-pneumonia)	216 (9.2%)
COPD exacerbation	52 (2.2%)
Cardiogenic shock/Acute cardiac syndromes	436 (18.6%)
Renal/Metabolic	1209 (51.4%)
Gastrointestinal/Hepatic	474 (20.2%)
Neurologic diagnoses	273 (11.6%)
Trauma/Burns	185 (7.9%)
Postoperative	151 (6.4%)
**Comorbidities, n (%)**	
Hypertension	873 (37.1%)
Hyperlipidemia	433 (18.4%)
Diabetes mellitus	824 (35.1%)
Obesity (BMI > 30)	722 (30.7%)
Ischemic heart disease	380 (16.2%)
Atrial fibrillation	299 (12.7%)
COPD	210 (8.9%)
Chronic kidney disease	194 (8.3%)
Cirrhosis	52 (2.2%)

[SOFA- Sequential organ failure assessment, COPD-chronic obstructive pulmonary disease]

### Cohort and exposure definition

**Fig. 2:**
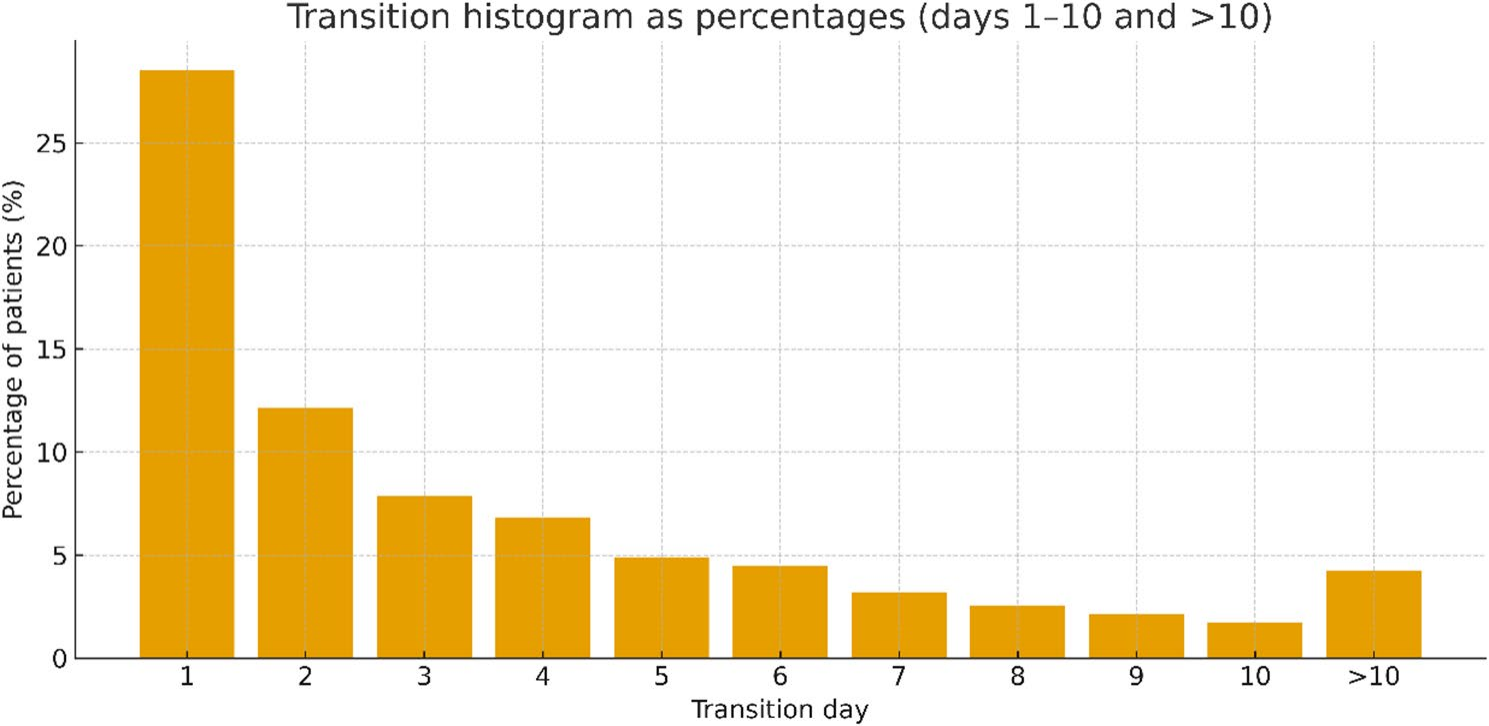
Distribution of metabolic transition days in the ICU cohort. Histogram showing the percentages of patients achieving catabolic-to-anabolic transition on each ICU day (days 1–10, with a final bin for > 10 days). Transition was defined by a ≥ 30% drop in insulin resistance index (IRI) sustained below 70% of peak for 24 h, accompanied by ≥ 2 of 8 physiologic recovery criteria (hemodynamic, inflammatory, and albumin markers). The majority of transitions occurred within the first 72 h, with a peak on day 1, and progressively fewer transitions observed thereafter

Of the 2,350 included patients, 2,209 (94%) fulfilled transition criteria. Most transitions occurred within the first week of ICU admission, with the distribution markedly front-loaded (peak on day 1 and a progressive decline thereafter; Fig. 2).

### Timing of transition

Transition timing was markedly front-loaded (Fig. 2). Approximately ~ 60% of transitions occurred by day 3, with the modal day at day 1 and a progressively smaller tail thereafter; a minority occurred > 10 days after admission. This pattern indicates that, in this iteration, the catabolic → anabolic transition usually occurs early during ICU stay.

### Survival by transition status

**Fig. 3:**
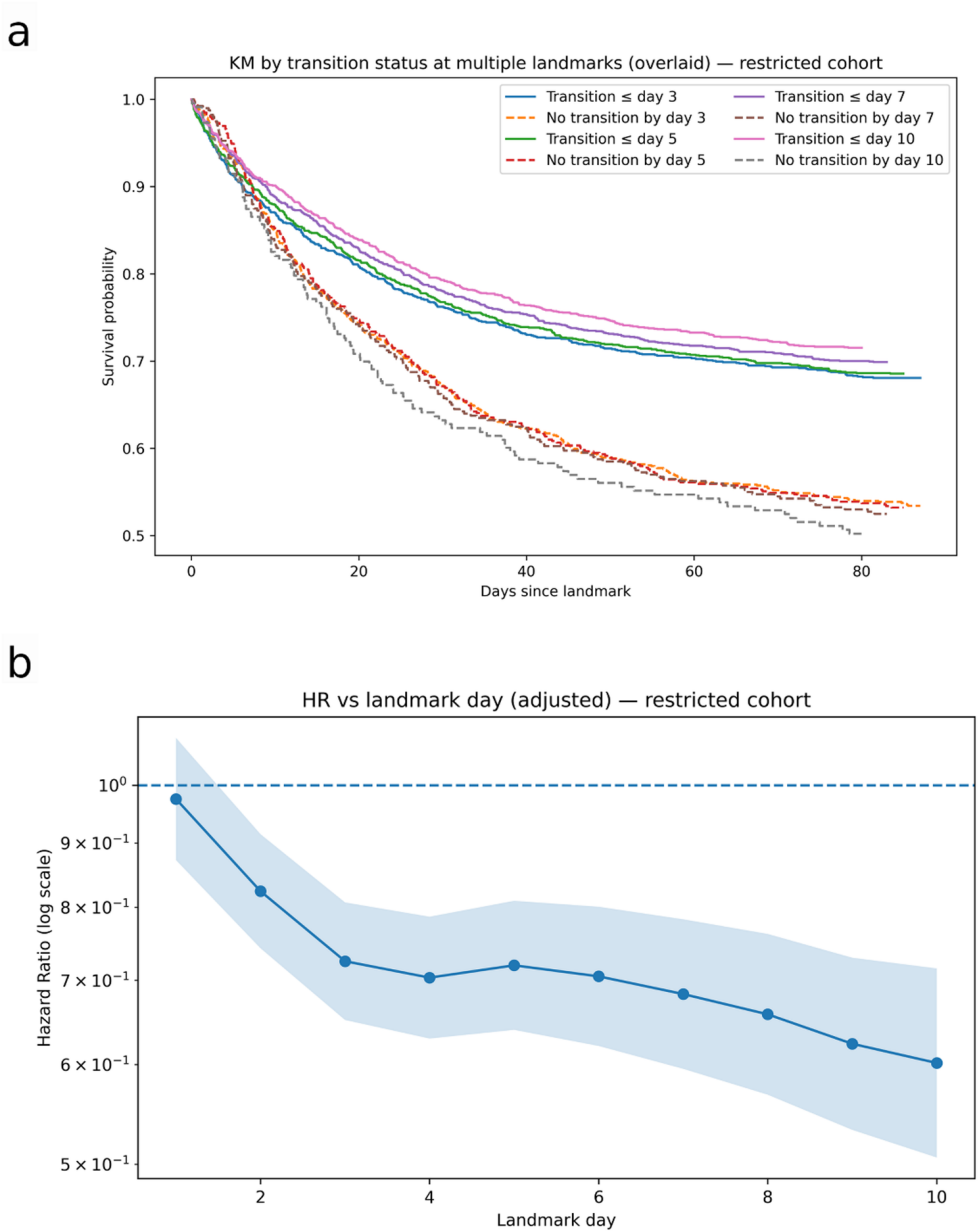
Transition versus mortality. **a. **Kaplan–Meier survival curves by transition status at multiple landmark time points. Survival probability is shown for patients stratified by whether they had achieved metabolic transition (solid lines) or not (dashed lines) by landmark days 3, 5, 7, and 10. At each landmark, patients who had transitioned exhibited consistently higher survival probabilities over the subsequent 90 days, with increasing separation of curves at later landmarks. **b. **Adjusted hazard ratios for 90-day mortality by transition status across landmark days. Hazard ratios (solid line, log scale) with 95% confidence intervals (shaded area) are shown for patients who achieved metabolic transition versus those who had not, at landmark days 1–10. Transition was defined by a ≥ 30% decline in insulin resistance index (IRI) with ≥ 2 physiologic recovery criteria. The protective association of transition strengthened progressively with later landmarks, with hazard ratios declining from ~ 0.98 at day 1 to ~ 0.60 by day 10, indicating lower mortality risk among patients who had transitioned.

Kaplan–Meier curves overlaid across landmarks (day 3, 5, 7, 10) showed consistent separation: at each landmark, patients who had already transitioned by the landmark had higher subsequent 90-day survival than those who had not yet transitioned. The magnitude of separation increased with later landmarks, in keeping with the growing fraction of patients who had transitioned and with the biological expectation that a sustained anabolic state associates with improved outcomes (Fig. 3A).

### Landmark Cox proportional hazards (90-day mortality)

Landmark cohorts ranged from 2,338 patients at day 1 to 2,015 at day 10, after excluding those who had died or were censored prior to each landmark. In multivariable landmark Cox models adjusted for age, sex, baseline SOFA score, admission diagnoses, and major comorbidities, metabolic transition by the respective landmark was consistently associated with lower 90-day mortality, with the magnitude of the protective association increasing at later landmarks. At day 1, no association was observed (HR 0.98, 95% CI 0.87–1.09), and a modest effect appeared at day 2 (HR 0.82, 95% CI 0.74–0.91). By day 3, transition status was associated with significantly reduced mortality (HR 0.72, 95% CI 0.65–0.81; *n* = 2,242; 848 events), with similar results at day 5 (HR 0.72, 95% CI 0.64–0.81; *n* = 2,165; 771 events). Stronger associations were seen at day 7 (HR 0.68, 95% CI 0.60–0.78; *n* = 2,094; 700 events) and day 10 (HR 0.60, 95% CI 0.51–0.72; *n* = 2,015; 621 events). Across days 1–10, the hazard ratio trajectory declined monotonically from ~ 0.98 to ~ 0.60 (Fig. 3B), indicating a progressively stronger survival advantage with earlier transition. When comparing transitioned versus non-transitioned patients overall, transition status was independently associated with improved survival, with an odds ratio for 90-day mortality of 0.57 (95% CI 0.40–0.80; *p* = 0.001). For more information – see supplement 2 and 3.

### Sensitivity to the iteration’s design choices

To evaluate the robustness of the model, we performed sensitivity analyses focusing on key design assumptions. This iteration deliberately excluded nutrition from the IRI denominator and applied a steroid base correction of 0.6; despite these stricter assumptions, the signal was consistent (KM separation and HR < 1 from day 3 onward). Requiring ≥ 2 of 8 ancillary parameters ensured that transition calls aligned with broader clinical recovery (hemodynamics, inflammation, albumin), which likely contributed to the robustness of the association.

### Nutrition before transition, overfeeding and mortality

**Fig. 4:**
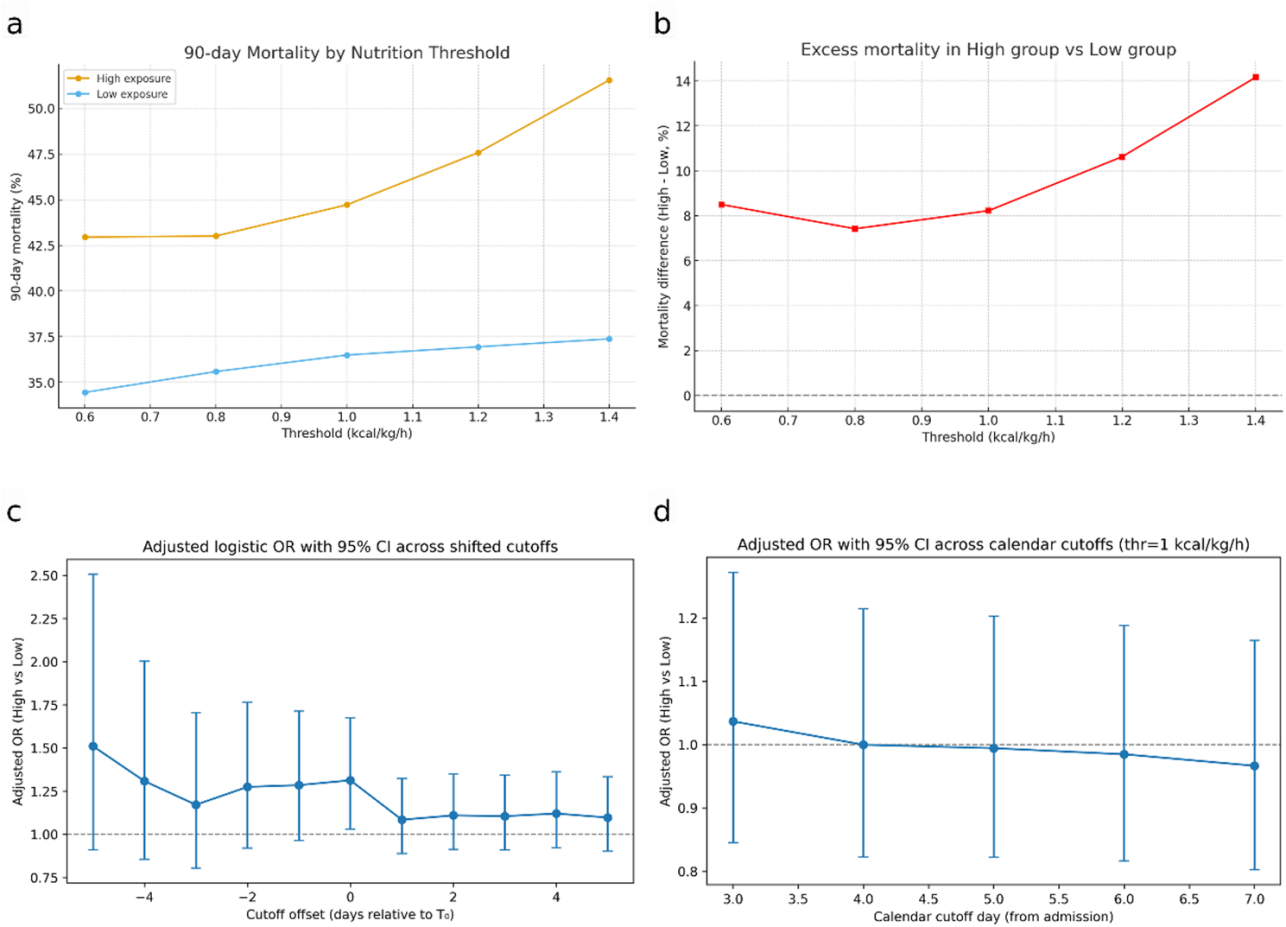
Association Between Pre-Transition Overfeeding and 90-Day Mortality Across Caloric Thresholds. **a.** Ninety-day mortality by exposure group across thresholds (0.6–1.4 kcal/kg/h). Mortality rates in the High nutrition group (≥ threshold for ≥ 24 h) and Low group (< threshold) are plotted for each cutoff. Mortality in the High group consistently exceeded that of the Low group, with divergence increasing at higher thresholds. **b.** Excess mortality (High–Low) across thresholds. Absolute mortality difference between High and Low groups, expressed as percentage points, plotted against the kcal/kg/h threshold. The mortality gap widened progressively from ~ 7–9% at 0.6–1.0 kcal/kg/h to ~ 14% at ≥ 1.4 kcal/kg/h, suggesting a dose–response relationship. **c**. Adjusted odds ratios (OR, 95% CI) for 90-day mortality comparing high versus low pre-transition intake when the cutoff defining the pre-transition window is shifted relative to the model-derived transition time (T₀). Mortality risk is greatest when the window precedes T₀ (negative offsets) and approaches neutrality thereafter. **d.** Adjusted OR (95% CI) using fixed calendar-day cutoffs from ICU admission (days 3–7) with a 1.0 kcal/kg/h threshold, showing no consistent association.

In adjusted analysis, patients receiving high pre-transition calories exposure (≥ 1.0 kcal/kg/h for ≥ 24 h before transition) had a significantly higher risk of death within 90 days compared with those who remained < 1.0 kcal/kg/h throughout the pre-transition phase (OR 1.25, 95% CI 1.01–1.55, *p* = 0.038). Sensitivity analyses across thresholds of 0.6, 0.8, 1.0, 1.2, and 1.4 kcal/kg/h demonstrated a consistent excess risk in the high calories group, with absolute mortality differences ranging from 7 to 9% at the lower thresholds to 14% at ≥ 1.4 kcal/kg/h, indicating a dose–response relationship (Fig. 4). For more information see supplement 4.

Across shifted cutoffs relative to the model-defined transition time (Fig. 4c), higher caloric intake before T₀ was associated with progressively greater 90-day mortality, with odds ratios exceeding 1.3 for several pre-transition windows. The association weakened after the transition, and statistical significance was reached only at the true T₀. In contrast, when using fixed calendar-day cutoffs from admission (Fig. 4d), no significant mortality difference was observed.

We also compared the calories intake at the 24 h period before versus after transition. The median intake increased slightly from 0.79 ± 0.88 to 0.88 ± 0.90 kcal/kg/h, corresponding to a mean paired change of + 0.09 ± 0.79 kcal/kg/h (95% CI 0.06–0.13), or ~ + 2.2 kcal/kg/day.

### Causal analysis

In order to estimate the potential causal effect of the catabolic-to-anabolic transition on 90-day mortality, doubly robust causal inference models were applied using inverse probability of treatment weighting (IPTW) and augmented inverse probability weighting (AIPW). After weighing, covariate balance was achieved across all major baseline characteristics (standardized mean differences < 0.05). The estimated average treatment effect (ATE) indicated a significant reduction in 90-day mortality among transitioned patients (ATE − 0.044, 95% CI − 0.065 to − 0.023, *p* < 0.001), corresponding to an absolute survival benefit of approximately 4.4%. The direction and magnitude of this causal effect were consistent across both IPTW and AIPW estimators, and sensitivity analyses suggested robustness to moderate unmeasured confounding (E-value 2.4; Rosenbaum Γ = 1.6).

## Discussion

In this large, retrospective cohort study, we developed and preliminarily evaluated a novel, multi-parameter model to identify the metabolic transition from catabolism to anabolism in critically ill patients. Our primary finding is that an earlier transition, as defined by a sustained drop in an insulin resistance index and concurrent improvement in physiological markers, is strongly and independently associated with lower 90-day mortality. This association strengthened with increased time from ICU admission, with a hazard ratio for mortality reaching 0.60 for patients who transitioned by day 10, suggesting a potential link between sustained metabolic recovery and survival. Patients who did not achieve metabolic transition experienced substantially higher 90-day mortality, underscoring the prognostic importance of this physiological shift.

Although sicker patients are expected to have higher mortality, the prognostic value of the transition model extended beyond baseline severity. Transition reflects a dynamic recovery process, not initial acuity, and the association with survival remained strong after adjustment for age, SOFA score, comorbidities, and diagnoses. Moreover, patients with similar initial metabolic derangements separated sharply by timing of transition, indicating that failure to recover—rather than baseline severity alone—drives much of the observed risk.

Transition timing showed a clear front-loaded pattern, with a greater proportion of patients transitioning earlier and progressively fewer transitioning later during the ICU course. This distribution reflects the heterogeneity of acute illness severity, the specific case mix of a given ICU, and the profound inter-patient heterogeneity in insulin resistance, inflammatory resolution, and metabolic capacity [26, 31, 32]. It also suggests that uniform calendar-based feeding strategies may not align with individual metabolic trajectories. Our findings indicate that some patients begin metabolic recovery well before the guideline-defined day 3–7 window, whereas others transition far later, highlighting limitations of a fixed-timeline approach. These results emphasize that current guidelines broad recommendations stem from the absence of validated physiologic markers [3, 4, 26, 31, 32, 36]. A transition-based framework may enable more individualized nutritional timing and dosing but requires further validation before influencing clinical practice.

The predominance of transition on day 1 suggests that for many patients, the IRI peak and early physiologic stabilization occur very rapidly after admission. This may reflect prior resuscitation in the emergency department, OR, or referring department/hospital, meaning the ICU ‘time zero’ does not always coincide with the onset of critical illness.

High pre-transition caloric exposure is independently associated with increased 90-day mortality, even after adjusting for age, SOFA score, sex, and admission diagnoses. The consistency of findings across multiple caloric thresholds, with progressively greater excess mortality at higher cutoffs, suggests a potential **dose–response relationship** and aligns with prior evidence that early overfeeding may be harmful [7, 17–20].

The finding that statistical significance was observed only at the model-defined transition time (T₀) supports the physiologic validity of this approach. Mortality risk from high caloric intake was greatest immediately before T₀ and diminished thereafter, indicating that the model identifies a true inflection point in metabolic recovery. In contrast, calendar-based cutoffs showed no association with outcome, emphasizing that nutritional readiness varies between patients and cannot be captured by fixed time-from-admission criteria.

Metabolic transition was not accompanied by a clinically meaningful change in caloric delivery. The median change in caloric intake across the transition window was only 2.2 kcal/kg/day, and more than half of patients experienced no change in feeding rates. This suggests the shift often occurs silently [36], unrecognized by clinicians. Importantly, this observation addresses a potential alternative explanation for our mortality findings: because caloric delivery did not systematically increase at the time patients met the transition criteria, the association between earlier transition and improved survival is unlikely to be explained simply by contemporaneous clinician-driven escalation of feeding in patients who appeared to be recovering. Rather, it highlights a potential care gap in which routine nutritional support is not dynamically adapted to the patient’s evolving metabolic state.

Taken together, the data support the hypothesis that the pre-transition metabolic state may not be suited for full caloric provision and suggest the rationale for a **restrictive feeding strategy before transition**, pending confirmation in prospective studies.

### Limitations

Several important limitations must be acknowledged. First, as an observational study, our findings demonstrate strong associations but cannot establish causality. It is plausible that the metabolic transition serves primarily as a marker of underlying recovery and is presumed as a surrogate to transition from catabolic to anabolic state.

Second, delivered kcal/kg/h may misrepresent true delivery because of interruptions, malabsorption, and unaccounted sources (e.g., propofol calories).

Third, the model was both developed and validated within a single academic medical center; generalizability to other institutions, patient populations, and clinical practices remain uncertain. External validation and complementary predictive modeling will therefore be essential.

### Future directions and implications

The present findings are hypothesis-generating and establish a foundation for future investigation. The immediate priority is prospective, multicenter validation to confirm accuracy, reproducibility, and clinical utility across diverse ICU populations. If validated, this trajectory-based model could serve as a stratification or enrichment tool for interventional trials, enabling researchers to test whether nutritional therapy titrated to an individual’s physiologic transition point-rather than an arbitrary calendar day-can improve outcomes.

## Conclusion

We developed and preliminary evaluated a novel, trajectory-based model that aims to identify a metabolic transition window in critically ill patients. In this large cohort, an earlier transition was strongly and independently associated with lower mortality risk, though this association may reflect broader recovery processes, presumed to be related to metabolic transition. Demonstrating increased mortality with overfeeding before transition emphasizes the clinical implication. This physiology-driven tool provides a preliminary, hypothesis-generating framework for future research aimed at potentially informing personalized nutritional therapy based on a patient’s evolving metabolic state.

## Supplementary Information

Supplementary Material 1

Supplementary Material 2

Supplementary Material 3

Supplementary Material 4

Supplementary Material 5

## Data Availability

The datasets used and/or analyzed during the current study are available from the corresponding author on reasonable request.
