# Supplementary material for: Detecting the metabolic transition to personalize nutritional timing: model development and preliminary validation in a large ICU cohort
Source: Crit Care. 2026 Feb 24;30:132. doi: 10.1186/s13054-026-05874-5 (PMC13037178; doi:10.1186/s13054-026-05874-5)
Supplement: Supplementary file 1 — Supplementary Material 1 [file 13054_2026_5874_MOESM1_ESM.docx]

Supplement 1: Sensitivity Analysis Protocol for the ICU Transition Analyzer

# **1. Objective**

The primary objective of this sensitivity analysis is to assess the robustness and stability of the metabolic transition definition. By systematically varying the core parameters that define the transition event, this analysis evaluates how the classification of patients (i.e., the number identified as having transitioned) changes in response to different analytical assumptions. The goal is to ensure that the model's findings are not an artifact of a single, arbitrarily chosen set of thresholds.

# **2. Methodology**

The analysis employs a grid search methodology. It constructs a multi-dimensional parameter space where each dimension represents a key model parameter. The analysis then iterates through every possible combination of the predefined parameter values, running the full transition detection algorithm for each unique configuration. The total number of patients who meet the transition criteria is recorded for each configuration, allowing for a comprehensive evaluation of the model's stability.

# **3. Analyzed Parameters**

The sensitivity analysis is performed on four key parameters that are central to the IRI calculation and the transition detection logic.

## **3.1. IRI Drop Fraction (iri_drop)**

- Definition: This parameter represents the required percentage decrease in the Insulin Resistance Index (IRI) from its observed peak value. It serves as the primary trigger for identifying a potential metabolic transition point. A transition is only considered after the patient's IRI falls below this threshold.
- Values Tested: 30%, 40%, 50%, 60%, 70%
- Rationale: To determine the model's sensitivity to varying magnitudes of improvement in insulin sensitivity. Testing a range of values helps identify whether the transition phenomenon is a gradual process or a discrete event linked to a specific degree of metabolic recovery.

## **3.2. Minimum Number of Criteria (min_criteria)**

- Definition: The minimum number of secondary clinical recovery markers that must be satisfied concurrently with the IRI drop to confirm a transition event. The model checks for up to eight corroborating markers related to inflammation, vasopressor support, and metabolic status.
- Values Tested: 2, 3, 4, 5
- Rationale: To evaluate how the stringency of the transition definition affects patient classification. A lower value represents a more liberal definition, while a higher value represents a more conservative and clinically specific definition. This analysis assesses the trade-off between sensitivity and specificity.

## **3.3. Nutrition Inclusion (nutrition_on)**

- Definition: A binary parameter (True/False) that determines whether the rate of caloric intake from enteral nutrition (normalized to kcal/kg/hr) is included in the denominator of the IRI formula.
- Values Tested: True (Nutrition is included), False (Nutrition is excluded, and the denominator defaults to 1)
- Rationale: The provision of nutrition is a significant external factor influencing glucose and insulin dynamics. This analysis assesses the impact of caloric support on the IRI calculation. It also tests the model's performance in scenarios where nutrition data may be unreliable or where the intent is to model insulin resistance independent of caloric intake.

## **3.4. Steroid Factor Base (steroid_base_high)**

- Definition: This parameter sets the baseline multiplier used to adjust the raw IRI for patients receiving high doses of corticosteroids (>200 mg hydrocortisone-equivalent in the prior 24 hours). This factor corrects for the known hyperglycemic effects of steroids.
- Values Tested: 0.4, 0.5, 0.6, 0.7
- Rationale: To test different clinical and physiological assumptions regarding the potency of iatrogenic hyperglycemia induced by high-dose steroids. A lower value assumes a stronger effect (requiring a larger downward correction of the IRI), while a higher value assumes a weaker effect. This parameter explores the model's sensitivity to different interpretations of corticosteroid impact.

## **Detailed Analysis of the Steroid Factor Calculation**

The steroid factor is a critical component that adjusts the IRI for the hyperglycemic effects of corticosteroid medications. The calculation is a multi-step process designed to standardize steroid potency and apply a dose-dependent correction.

Step 1: Potency Standardization

First, all steroid administrations (hydrocortisone, methylprednisolone, dexamethasone) are converted to a standardized unit: the hydrocortisone equivalent dose. This is achieved using established glucocorticoid potency ratios:

- Hydrocortisone: 1 mg = 1.0 mg hydrocortisone equivalent
- Methylprednisolone: 1 mg = 5.0 mg hydrocortisone equivalent
- Dexamethasone: 1 mg = 26.7 mg hydrocortisone equivalent

Step 2: Time-Varying Dose Calculation

For each glucose measurement timepoint, the model calculates the total hydrocortisone equivalent dose administered in the preceding 24-hour window. This creates a dynamic, time-varying measure of the patient's total steroid exposure.

Step 3: Tiered Factor Application

The total 24-hour hydrocortisone equivalent dose is used to assign a specific correction factor based on a three-tiered system. This system is directly modulated by the steroid_base_high parameter from the sensitivity analysis.

| Tier | 24-hr Hydrocortisone Equivalent | Correction Factor Formula | Interpretation |
| --- | --- | --- | --- |
| 1 | > 200 mg (Very High Dose) | steroid_base_high | Represents the maximum assumed steroid effect, resulting in the most significant IRI reduction. |
| 2 | 100 - 200 mg (High Dose) | steroid_base_high + 0.2 | Represents a strong but sub-maximal steroid effect, leading to a moderate IRI reduction. |
| 3 | < 100 mg (Low/Moderate/No Dose) | steroid_base_high + 0.25 | Represents the minimal steroid effect, resulting in the smallest IRI reduction. |

## **3.5 Insulin added factor**

The IRI formula included a constant of 0.5 U/h added to the insulin infusion rate. This choice was pragmatic, preventing the index from reaching zero when exogenous insulin was paused and reflecting a basal endogenous insulin secretion. To test whether the choice of the added insulin constant influenced results, we repeated the analysis with alternative constants of 0.1 and 1.0 U/h (reference 0.5 U/h), while keeping all other parameters fixed (30% IRI drop, ≥2 supportive criteria, steroid correction, nutrition OFF).

## **3.6 Criteria thresholds**

To evaluate the robustness of the basic transition model ( ≥30% decrease in IRI from its individual peak, a sustained drop for ≥24 hours, and the fulfillment of **≥**2 of 8 supportive criteria), we also performed a post hoc grid-based sensitivity analysis in which we varied the percentage-drop thresholds required for each supportive physiological marker. Lactate, CRP, neutrophils %, and all vasopressors were tested at 20%, 30%, and 40% reductions from peak; WBC at 30%, 40%, and 50%; and albumin at 0%, 5%, 10%, and 15% relative rise compared with the previous measurement. In accordance with the analytic code, a single vasopressor cutoff was applied uniformly to norepinephrine, adrenaline, and vasopressin, although their individual contribution as qualifying criteria was still recorded separately.

Each combination of marker-specific thresholds was applied to the full cohort using the same IRI trajectories, peak detection, and transition logic as the base model. For every configuration, we extracted the overall transition detection rate, transition timing, mortality stratification, and the frequency with which each criterion contributed to meeting the supportive-criteria requirement.

# **Sensetivity analysis results**

## **Step 1 – stability analysis**


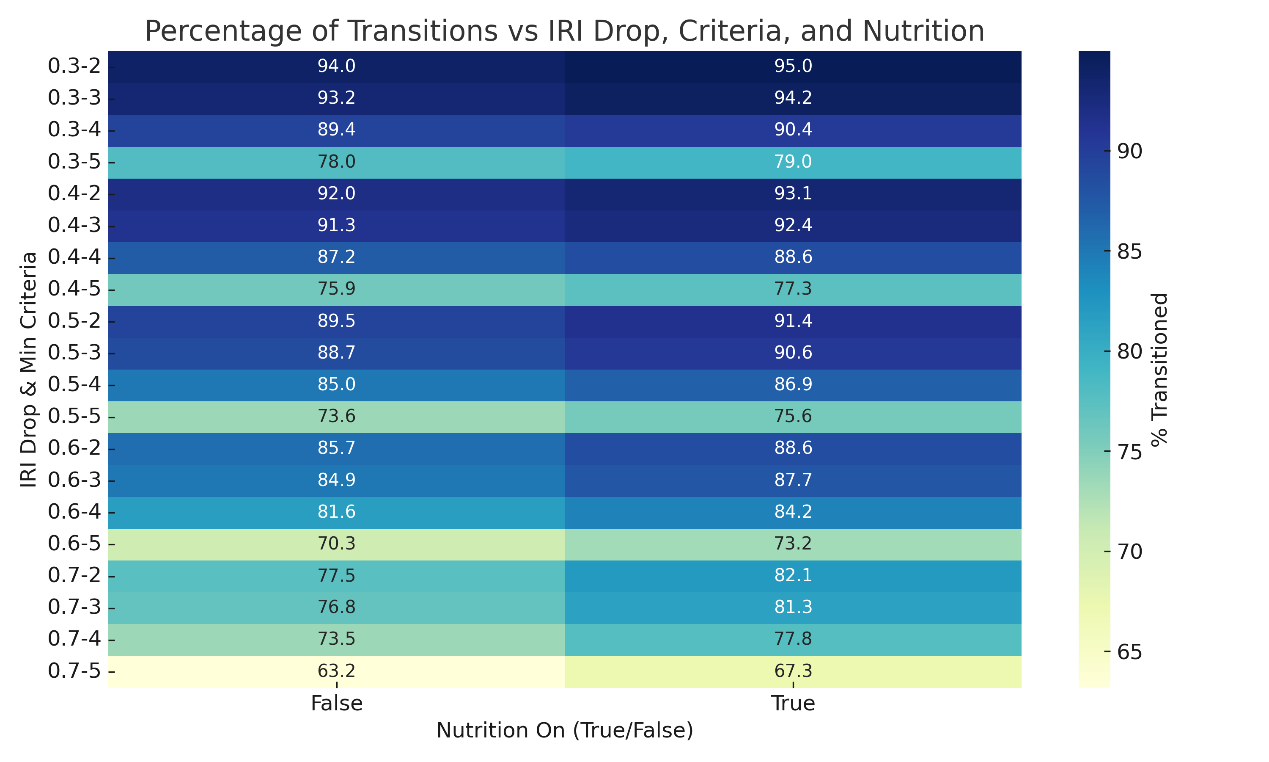
 **Figure S1:** Heatmap of the percentage of transitioned patients across different thresholds

The heatmap in Figure 1 provides a two-dimensional visualization of the sensitivity analysis results. It illustrates the percentage of the eligible patient cohort that meets the criteria for metabolic transition across the primary model parameters. The x-axis represents the required IRI drop threshold, ranging from 30% to 70%. The y-axis represents the Minimum Criteria required to confirm a transition, ranging from 2 to 5. The color intensity corresponds to the percentage of patients who transitioned, with warmer colors (yellow) indicating a lower percentage and cooler colors (dark blue) indicating a higher percentage.

The most significant change in the percentage of patients identified as transitioned is consistently driven by the increase in minimum criteria from 4 to 5, and the magnitude of this change is remarkably consistent across all IRI drop levels. For example, under the "Nutrition On" condition, this increase in stringency causes the transition percentage to fall by 11.4 points at the 30% IRI drop threshold (from 90.4% to 79.0%) and by 11.3 points at the 60% threshold (from 84.2% to 73.2%). This consistent, dose-dependent response to increasing clinical stringency indicates that the model behaves predictably across the entire tested parameter space.

**Nutrition On vs. Off**

The heatmap clearly shows that including nutritional intake in the Insulin Resistance Index (IRI) calculation has a consistent effect on the model's output.

- **Higher Transition Rates:** For every combination of IRI drop and minimum criteria, including nutrition results in a higher percentage of patients being identified as transitioned compared to when nutrition is excluded.
- **Similar Stability Profile:** The model's responsiveness to changes in other parameters remains very similar whether nutrition is included or not. For instance, the sharpest drop in transition percentage (when increasing the minimum criteria from 4 to 5) is of a nearly identical magnitude in both the "True" and "False" columns. This indicates that while the absolute percentages change, the model's structural stability isn't dramatically altered by the nutrition switch based on this visual evidence.

## **Step 2: Clinical Face Validity**

The primary objective of this step was to apply clinical judgment to select the most plausible model from the stable configurations identified in the initial analysis. A rigorous clinical target was set, requiring the chosen model to identify over 90% of the patient cohort as having transitioned.

## **Step 3 : Outcome analysis**

We performed a landmark Cox analysis of 90-day mortality at days 3, 7, and 10 post-ICU admission. For each iteration we defined “transition achieved” according to the prespecified rule (IRI drop threshold dX, number of concurrent criteria k, nutrition handling nut, and steroid factor st). Exposure at a given landmark was coded as transitioned on/before the landmark vs not yet transitioned. The time origin was the landmark; follow-up ended at death or 90 days. We adjusted for age, sex, and day-1 SOFA. Fits used a penalized, robust Cox proportional hazards model (lifelines), with automatic fall-back and safeguards when exposure was nearly constant.

## **Objective for choosing a primary iteration**

We sought a single specification that balances:

1. **Effect magnitude** (lower HR),
2. **Cross-day consistency/availability** (estimates at days 3, 7, and 10 all <1 with narrow CIs), and
3. **Positivity/robustness** (a non-trivial unexposed group at each landmark).

We therefore ranked iterations by: number of days with **beneficial & significant** HRs (HR<1 with 95%CI entirely <1), **mean log(HR)** across days, total events, and usable n. We also monitored the **unexposed fraction** (100 − transition%) to avoid extreme exposure imbalance.

# **What the data showed**

For the **d30_k2** family (30% IRI drop, 2 supportive parameters):

- **nut1 (nutrition ON)** — best early HRs but very small control group
  - **d30_k2_nut1_st0.6**:
    - Day 3: **HR 0.696** (0.625–0.776), n=2242, transition **95.0%**
    - Day 7: **HR 0.685** (0.594–0.791), n=2094, transition **95.0%**
    - (Day 10 was not reliably available/estimable in our export for this exact setting.)
  - Interpretation: slightly stronger benefit at Day 3; Day 7 essentially ties the nut0 counterpart. However, ≈95% transitioned by Day 7 leaves very few controls, increasing sensitivity to model choice and missingness and limiting generalizability.
- **nut0 (nutrition OFF)** — nearly the same early HRs, plus strong Day-10 and slightly better balance.
  - **d30_k2_nut0_st0.6**:
    - Day 3: **HR 0.739** (0.663–0.823), n=2242, transition **94.0%**
    - Day 7: **HR 0.681** (0.595–0.780), n=2094, transition **94.0%**
    - Day 10: **HR 0.597** (0.502–0.709), n=2015, transition **94.0%**
  - Interpretation: virtually identical Day-7 performance to nut1, excellent Day-10 benefit, and a slightly larger unexposed group (~6% vs ~5%). The availability of a stable, clearly <1 HR at Day 10 improves cross-day consistency and reduces the risk that results hinge on a single timepoint. As sensitivity analyses demonstrated consistent results with and without nutrition, we selected the nut0 iteration to avoid confounding from caregiver-dependent nutritional practices

Other balanced contenders (kept as additional sensitivity specifications):

- **d50_k2_nut0_st0.6** (≈10–11% unexposed):
  - Day 3: **0.780** (0.701–0.868); Day 7: **0.728** (0.640–0.827); Day 10: **0.693** (0.593–0.810).
  - Rationale: preserves a strong effect while improving positivity.
- **d70_k2_nut0_st0.4** (≈22% unexposed):
  - Day 3: **0.839** (0.751–0.936); Day 7: **0.786** (0.699–0.883); Day 10: **0.779** (0.684–0.889).
  - Rationale: most conservative (largest control group), with stable benefit across days.

Across all top rows, varying the steroid factor (st 0.4–0.7) did not materially change rankings; st=0.6 was consistently competitive.

## **Insulin added factor**

Transition rates, median transition times, and the association with 90-day mortality were virtually identical across all three specifications. Although higher constants yielded slightly smoother IRI trajectories (lower coefficients of variation), the protective effect of transition on survival was robust to the exact constant applied. These findings support the use of 0.5 U/h as a physiologically plausible and stable choice, while demonstrating that the overall conclusions are not sensitive to this modeling assumption.

## **Criteria thresholds**

Across all tested threshold combinations, the transition-detection model remained **highly robust**. Transition rates varied only minimally (≈93–94%), and the median transition time shifted by only 0.1–0.2 days, even when applying much stricter percentage-drop requirements to WBC, CRP, neutrophils, lactate, vasopressors, or larger albumin-rise thresholds. As expected, tighter cutoffs reduced the frequency with which individual markers qualified as supportive criteria—particularly albumin at ≥10–15% rise and WBC at ≥50% drop—but these adjustments **did** not meaningfully change whether a transition was detected or when it occurred. The very small shifts in timing indicate that transition determination is **driven primarily by the IRI trajectory**, with supportive criteria providing confirmation rather than dictating the transition point. Mortality separation between transitioned and non-transitioned patients remained consistent across all configurations. Overall, the transition definition showed stable performance and strong resilience to wide variations in marker-specific drop thresholds.

# **Final choice and justification**

We selected **d30_k2_nut0_st0.6** as the **primary** iteration because it best met our composite objective:

- **Effect:** HRs substantially below 1.0 at all landmarks, with tight CIs—particularly strong at Day 10 (HR 0.597, 95%CI 0.502–0.709).
- **Consistency:** Beneficial and available at Days 3, 7, and 10 (nutrition on lacked a robust Day-10 estimate in our export).
- **Positivity/robustness:** High transition prevalence in both nutrition on and nutrition off, but nutrition off retains a slightly larger unexposed group at each landmark, making the comparison more stable and less model-sensitive.

In short, although nutrition on wins marginally at Day 3, nutrition off offers comparable Day-7 performance, strong Day-10 evidence, and better balance—hence its selection as the primary specification.


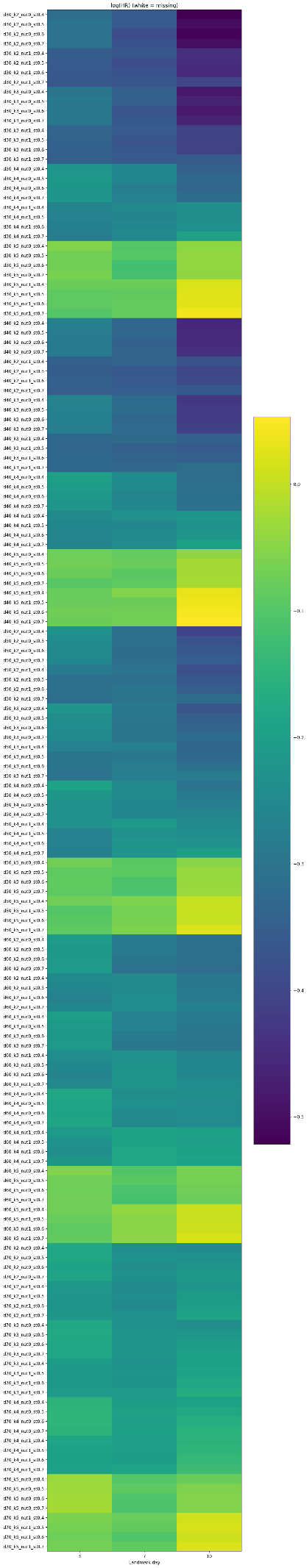


**Figure S2. Heatmap of model iteration analyses for 90-day mortality.**
Each row represents a distinct model configuration, varying by insulin resistance index (IRI) drop threshold, minimum number of supporting criteria, and inclusion of nutrition or steroid correction. Columns correspond to landmark days (3, 7, and 10 days from ICU admission). Colors indicate the log hazard ratio (log[HR]) for the association between catabolic-to-anabolic transition and 90-day mortality, with purple/blue denoting lower risk and green/yellow indicating higher risk. This visualization highlights how the estimated mortality effect of transition varies across analytic assumptions and timepoints.


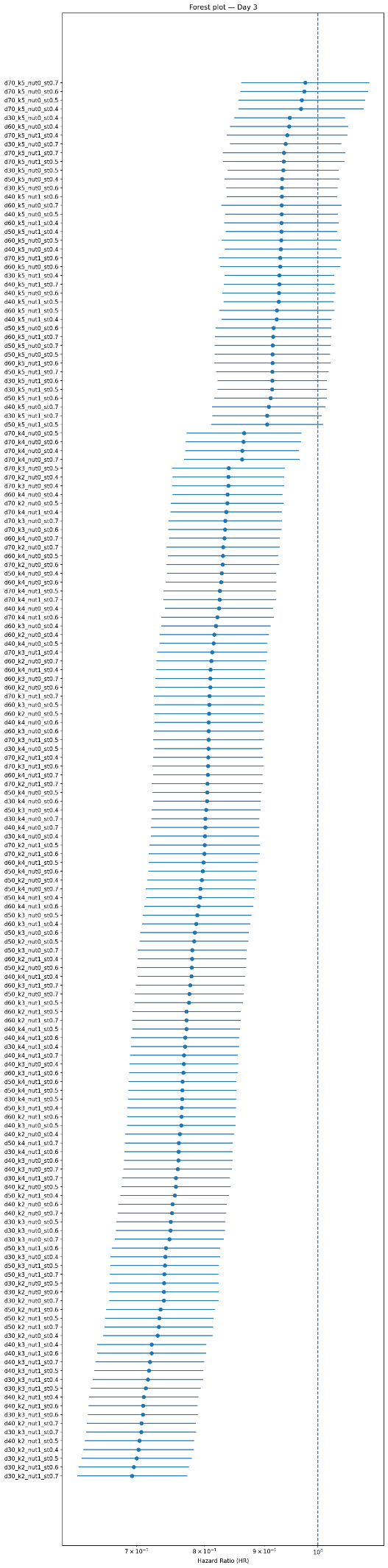


**Figure S3. Forest plot of model iterations for Day 3 landmark analysis.**
Each row represents a distinct analytic configuration varying by insulin resistance index (IRI) drop threshold, minimum number of criteria, and correction for nutrition or steroids. Points indicate hazard ratios (HR) for the association between transition by Day 3 and 90-day mortality, with horizontal lines denoting 95% confidence intervals. The vertical dashed line marks the null effect (HR = 1). Most model specifications consistently demonstrate HRs below 1, indicating a lower mortality risk among patients who transitioned early, although the magnitude of effect varies across analytic assumptions.


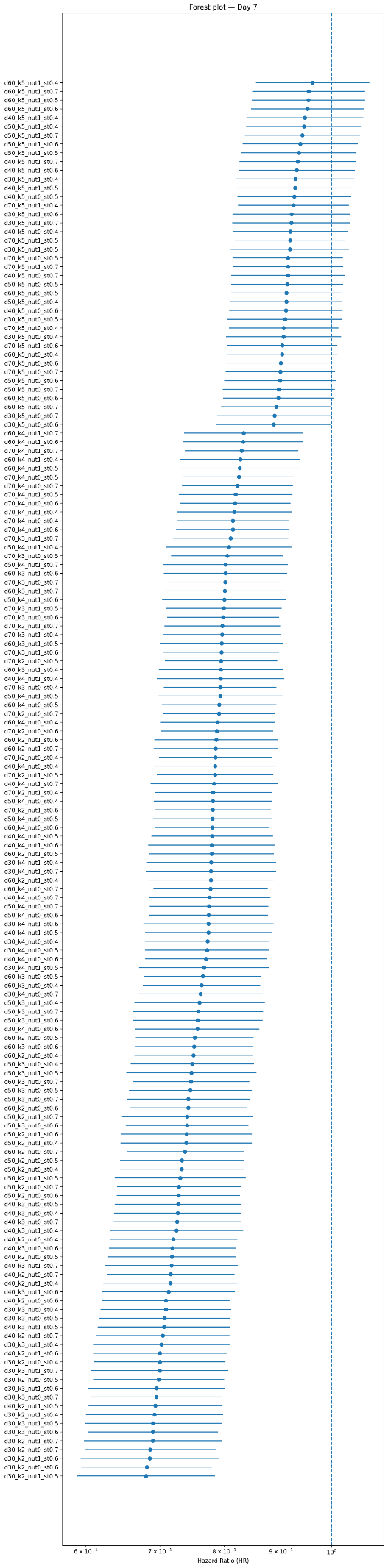


**Figure S4. Forest plot of model iterations for Day 7 landmark analysis.**
Each row depicts a distinct analytic configuration, varying by insulin resistance index (IRI) drop threshold, number of criteria required, and corrections for nutrition or steroid exposure. Dots represent hazard ratios (HR) for the association between achieving transition by Day 7 and 90-day mortality, with horizontal lines showing 95% confidence intervals. The vertical dashed line indicates the null effect (HR = 1). Across most specifications, HRs remain consistently below 1, suggesting that patients who transitioned by Day 7 experienced lower mortality, though the strength of association varies depending on model assumptions.


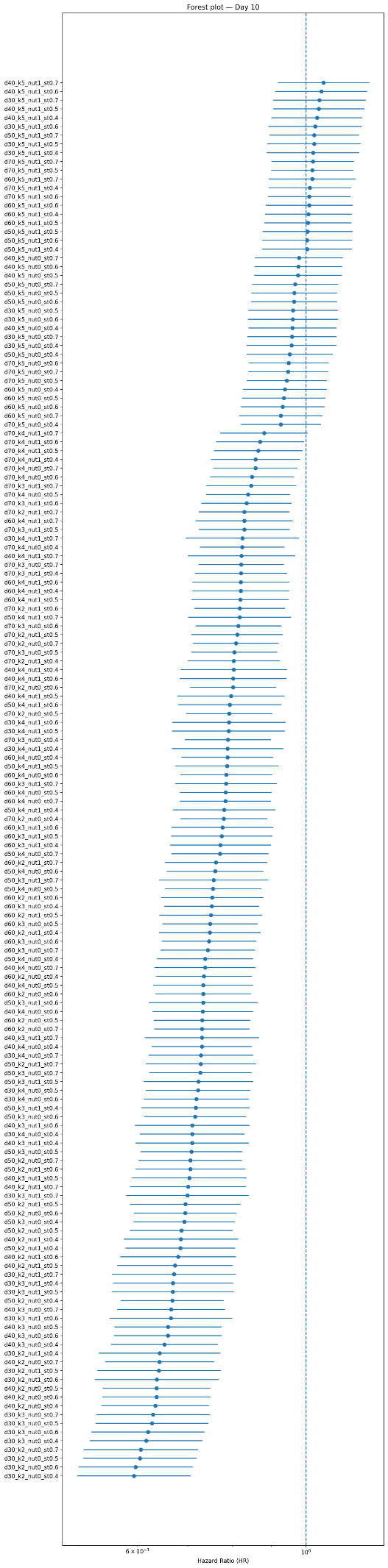


**Figure S5. Forest plot of model iterations for Day 10 landmark analysis.**
Each row depicts a distinct analytic configuration, varying by insulin resistance index (IRI) drop threshold, number of criteria required, and corrections for nutrition or steroid exposure. Dots represent hazard ratios (HR) for the association between achieving transition by Day 10 and 90-day mortality, with horizontal lines showing 95% confidence intervals. The vertical dashed line indicates the null effect (HR = 1). Across most specifications, HRs remain consistently below 1, suggesting that patients who transitioned by Day 10 experienced lower mortality, though the strength of association varies depending on model assumptions.
