# Supplementary material for: Detecting the metabolic transition to personalize nutritional timing: model development and preliminary validation in a large ICU cohort
Source: Crit Care. 2026 Feb 24;30:132. doi: 10.1186/s13054-026-05874-5 (PMC13037178; doi:10.1186/s13054-026-05874-5)
Supplement: Supplementary file 2 — Supplementary Material 2 [file 13054_2026_5874_MOESM2_ESM.docx]

Supplement 2 : IRI Trajectory Analysis by Transition Timing

# Methods

## Transition groups

Patients were stratified by transition timing using inclusive cut-points unless otherwise specified: ≤3 d (3), >3–5 d (5), >5–7 d (7), >7–10 d (10), and never (>10 d or missing).

## Trajectory aggregation

IRI values were aggregated in regular time bins to form group trajectories. For each (time bin × group) we computed the median and interquartile range (IQR).

## Confirmatory analyses

AUC(0–X d): Per patient, area under IRI vs time using the trapezoidal rule. Group differences assessed via Kruskal–Wallis; pairwise contrasts via Mann–Whitney with Holm correction. Effect sizes reported as Cliff’s δ.

Slopes (0–X d): Per-patient slope of IRI vs time using ordinary least squares (or robust Theil–Sen when selected). Group differences via Kruskal–Wallis and Holm-adjusted pairwise Mann–Whitney. A within‑patient fixed‑effects common slope by group is also provided in-app.

# Results

## AUC(0–20 d) by group

Global test: Kruskal–Wallis across groups (n_groups=5): H = 262.775, p = 1.15e-55.

## Per‑patient slopes (0–20 d) by group

Global test: Kruskal–Wallis across groups (n_groups=5): H = 917.731, p = 2.4e-197.

## Interpretation

Directionality: Median AUC increases with later transition timing (3 < 5 < 7 < 10 < never), consistent with higher cumulative insulin resistance when transition is delayed/absent.

Dynamics: Per‑patient slopes are most negative for early transition groups, indicating faster resolution of insulin resistance; late/never groups are flatter or positive.

Statistical evidence: Both AUC and slope distributions differ strongly across groups (Kruskal–Wallis p-values ≪ 0.001). Pairwise contrasts identify the largest gaps between early (e.g., 3 d) and late/never groups.

## Robustness & Sensitivity

Findings remain qualitatively unchanged under

1. inclusive vs strict cut-points,
2. time-binning choices (0.25–2 d),
3. trimming of extreme IRI values,
4. minimum measurement thresholds per patient, and
5. robust slope estimation via Theil–Sen in place of OLS.

# Supplementary Figures


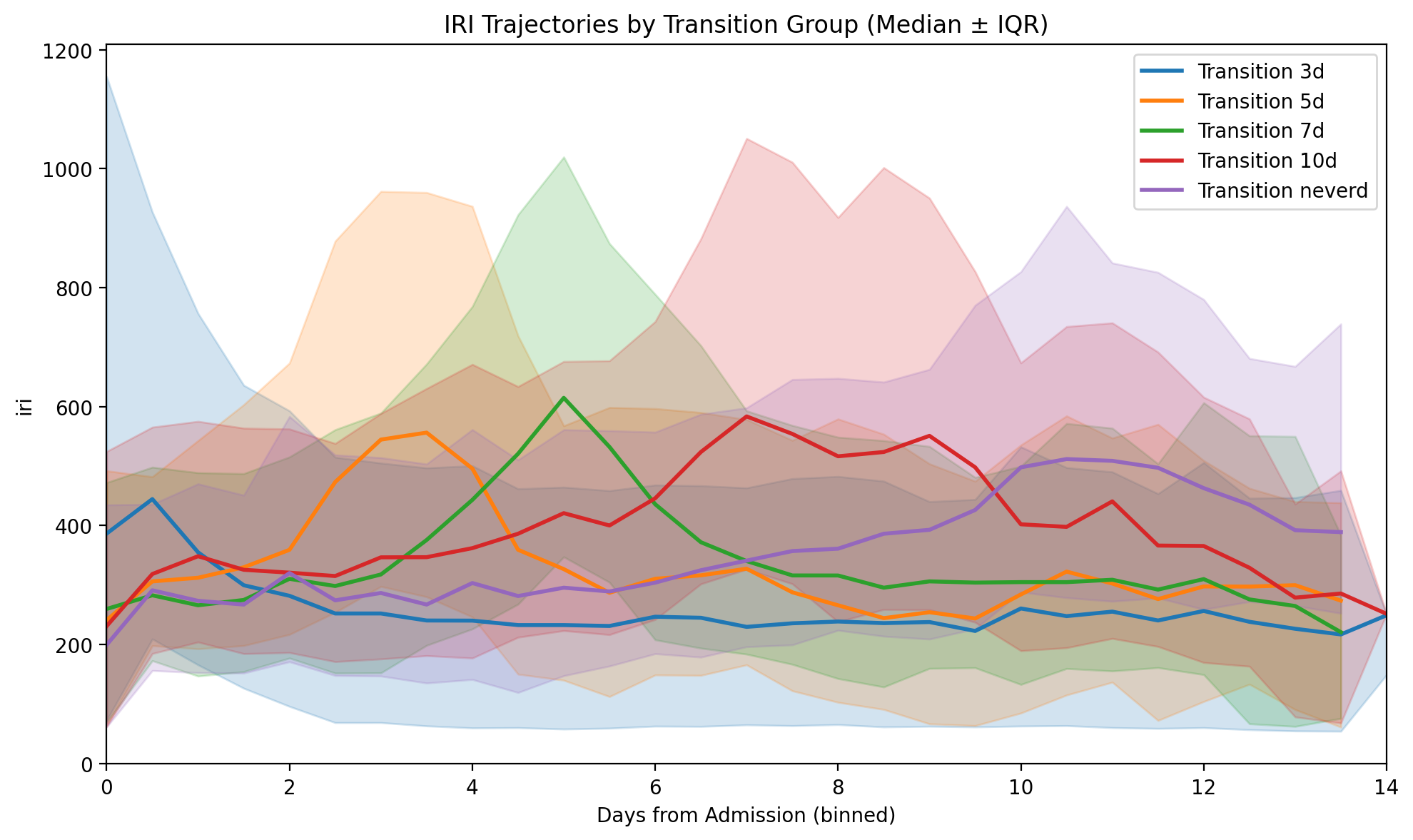


**Figure S1.** Insulin Resistance Index (IRI) trajectories by transition group.
Median IRI values (lines) with interquartile ranges (shaded areas) are shown from ICU admission through day 14, stratified by transition timing: Day 3 (blue), Day 5 (orange), Day 7 (green), Day 10 (red), and never transitioned (purple). Patients who transitioned earlier demonstrated higher initial IRI peaks followed by a more rapid decline, whereas those with delayed or absent transition exhibited persistently elevated or rising IRI values. This pattern supports the concept that early transition from catabolic to anabolic state is associated with faster resolution of insulin resistance.
