# Supplementary material for: Detecting the metabolic transition to personalize nutritional timing: model development and preliminary validation in a large ICU cohort
Source: Crit Care. 2026 Feb 24;30:132. doi: 10.1186/s13054-026-05874-5 (PMC13037178; doi:10.1186/s13054-026-05874-5)
Supplement: Supplementary file 3 — Supplementary Material 3 [file 13054_2026_5874_MOESM3_ESM.docx]

Supplement 3: Mortality by Metabolic Transition Status

This supplement summarizes mortality differences between patients who achieved a metabolic transition (defined by a ≥30% post‑peak IRI drop with persistence and 2 supportive criteria) and those who did not.

# Mortality in relation to transition

Among 2,350 ICU patients, 2,209 (94.0%) achieved a metabolic transition and 141 (6.0%) did not. Landmark Kaplan–Meier analyses at 3, 7, and 10 days demonstrated consistent separation in survival curves, with higher survival in the transitioned group (Figures S1–S3). In logistic regression, transition status was associated with significantly lower 90-day mortality (OR 0.57, 95% CI 0.40–0.80; p=0.001).


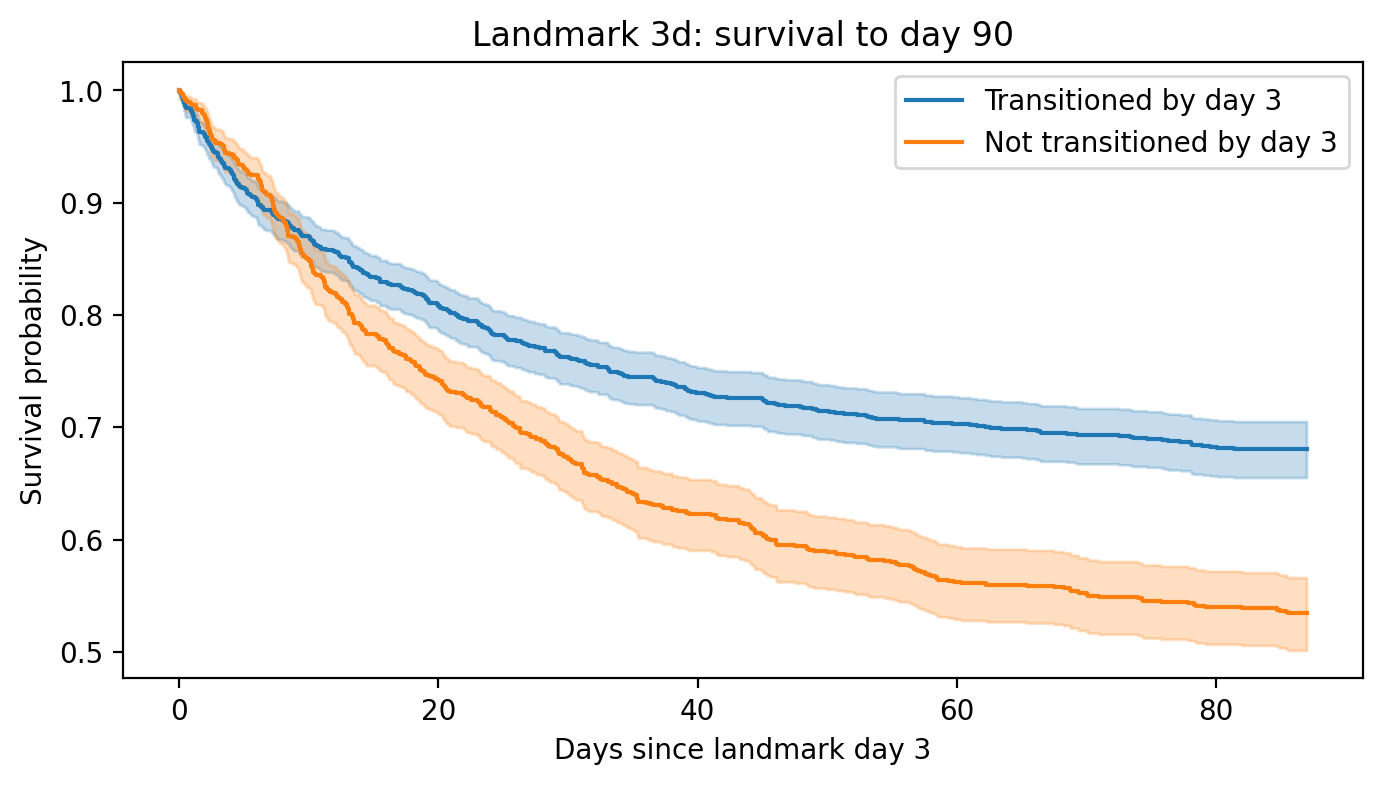


**Figure S1.** Kaplan–Meier survival curves at a 3-day landmark. Patients who had achieved a metabolic transition by day 3 are compared with those who had not. Transitioned patients demonstrate higher survival through 90 days.


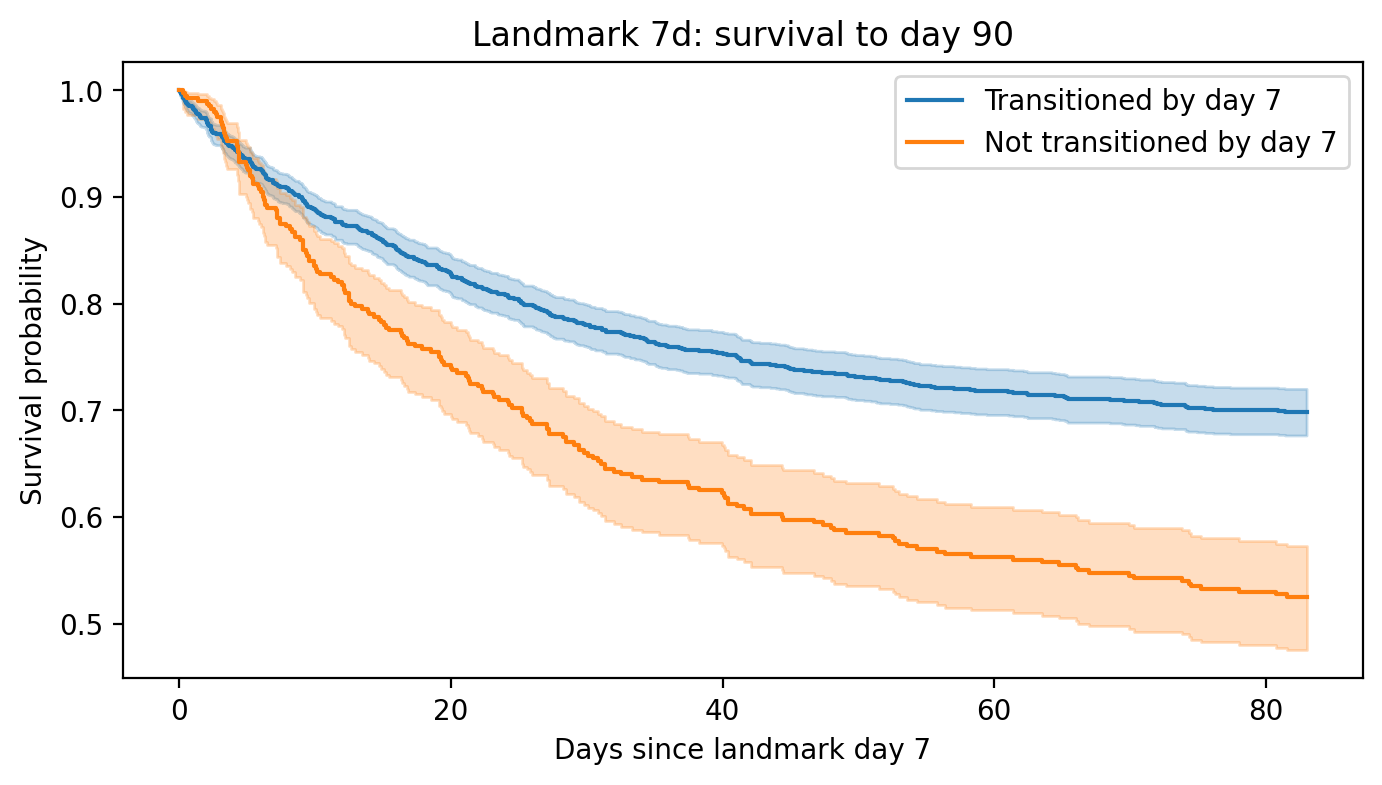


**Figure S2.** Kaplan–Meier survival curves at a 7-day landmark. Survival separation persists between transitioned and non-transitioned patients, with consistently lower mortality in the transitioned group.


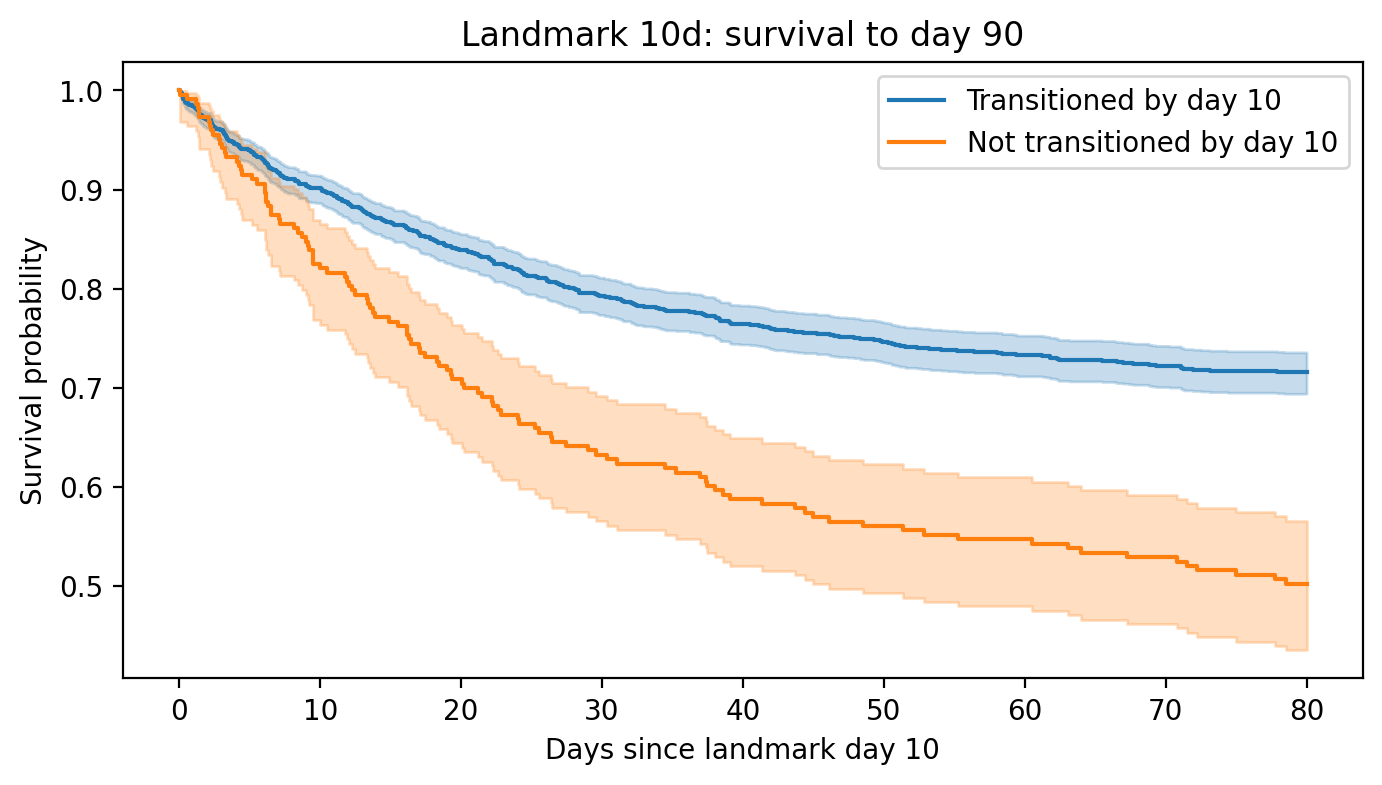


**Figure S3.** Kaplan–Meier survival curves at a 10-day landmark. By this later landmark, the divergence in 90-day survival remains evident, supporting the robustness of the association between transition and improved outcomes.

When comparing transitioned and non-transitioned patients, baseline comorbidities and admission diagnoses were largely similar (Table S1). Admission SOFA scores showed only modest differences between groups, with median values of 12 in both the early (≤5d) and late (>5d) transition groups, and 11 in the never-transitioned group. Despite this, mortality was highest among patients who never transitioned (54% at 90 days) compared with those who transitioned early (38%) or late (48%).

Mechanical ventilation was highly prevalent in all groups, but with important nuances: the late transition group had the highest prevalence (>90%), the early transition group slightly lower (~81%), and the never-transitioned group the lowest (~78%). Thus, the group with the highest mortality paradoxically showed somewhat lower use of mechanical ventilation and slightly lower SOFA scores at baseline. Stratification of the never-transitioned group by admission SOFA demonstrated that mortality increased stepwise with higher SOFA (Table S3): patients with SOFA ≥15 had early mortality exceeding 30% within 5 days and over 50% by 90 days, while those with SOFA ≤8 had far lower early mortality and ~40% mortality by 90 days. These findings indicate that the apparent paradox in the aggregate data reflects heterogeneity within the never-transitioned group: some patients died extremely early, before prolonged organ support could be instituted, while others survived longer but failed to demonstrate metabolic recovery.

| Table S1: Comparison of Admission Diagnoses and Comorbidities Between Transitioned and Non-Transitioned Patients | | | | |
| --- | --- | --- | --- | --- |
| p-value | Never | Late (>5d) | Early (<5d) | Characteristic |
| 0.071 | 63.2 [51.4, 71.8] | 65.4 [51.2, 72.7] | 63.2 [49.5, 71.3] | Age (years) |
| 0.022 | 11.0 [9.0, 14.0] | 12.0 [10.0, 15.0] | 12.0 [9.0, 14.0] | SOFA score at admission |
| 0.000 | 6.9 [3.6, 17.6] | 15.1 [10.7, 22.7] | 6.9 [4.0, 12.9] | ICU length of stay (days) |
| 0.178 | 91/141 (64.5%) | 304/476 (63.9%) | 1036/1733 (59.8%) | Male sex |
| 0.000 | 110/141 (78.0%) | 433/476 (91.0%) | 1402/1733 (80.9%) | Mechanical ventilation |
| 0.138 | 65/141 (46.1%) | 237/476 (49.8%) | 774/1733 (44.7%) | Sepsis |
| 0.164 | 55/141 (39.0%) | 201/476 (42.2%) | 649/1733 (37.4%) | Septic shock |
| 0.384 | 43/141 (30.5%) | 173/476 (36.3%) | 587/1733 (33.9%) | Acute kidney injury |
| 0.002 | 43/141 (30.5%) | 174/476 (36.6%) | 486/1733 (28.0%) | Pneumonia |
| 0.415 | 11/141 (7.8%) | 37/476 (7.8%) | 108/1733 (6.2%) | Trauma (MVA) |
| 0.505 | 7/141 (5.0%) | 17/476 (3.6%) | 55/1733 (3.2%) | Pancreatitis |
| 0.345 | 4/141 (2.8%) | 19/476 (4.0%) | 47/1733 (2.7%) | Cardiogenic shock |
| 0.336 | 3/141 (2.1%) | 15/476 (3.2%) | 35/1733 (2.0%) | Burns |
| 0.063 | 60/141 (42.6%) | 193/476 (40.5%) | 620/1733 (35.8%) | Hypertension |
| 0.356 | 50/141 (35.5%) | 180/476 (37.8%) | 594/1733 (34.3%) | Diabetes mellitus |
| 0.562 | 41/141 (29.1%) | 138/476 (29.0%) | 543/1733 (31.3%) | Obesity (BMI >30) |
| 0.094 | 26/141 (18.4%) | 104/476 (21.8%) | 303/1733 (17.5%) | Hyperlipidemia |
| 0.289 | 19/141 (13.5%) | 87/476 (18.3%) | 274/1733 (15.8%) | Ischemic heart disease |
| 0.912 | 34/141 (24.1%) | 114/476 (23.9%) | 401/1733 (23.1%) | Smoking history |
| 0.08 | 26/141 (18.4%) | 64/476 (13.4%) | 209/1733 (12.1%) | Atrial fibrillation |
| 0.066 | 7/141 (5.0%) | 35/476 (7.4%) | 168/1733 (9.7%) | COPD |
| 0.363 | 9/141 (6.4%) | 46/476 (9.7%) | 139/1733 (8.0%) | Chronic kidney disease |
| 0.044 | 0/141 (0.0%) | 16/476 (3.4%) | 36/1733 (2.1%) | Cirrhosis |

| Table S2: Comparison of Mortality Timing (5-Day, 30-Day, and 90-Day) Across Early, Late, and Never Transition Groups | | | | | | | |
| --- | --- | --- | --- | --- | --- | --- | --- |
| Group | **N patients** | **SOFA median [IQR]** | **N deaths** | **Death time median [IQR]** | **Deaths <5d** | **Deaths ≤30d** | **Deaths ≤90d** |
| Early (≤5d) | 1,733 | 12.0 [9–14] | 1,055 | 35.6 [9.0–361.0] | 158 (9.1%) | 492 (28.4%) | 653 (37.7%) |
| Late (>5d) | 476 | 12.0 [10–15] | 318 | 36.6 [16.4–159.2] | 0 (0.0%) | 136 (28.6%) | 227 (47.7%) |
| Never | 141 | 11.0 [9–14] | 105 | 19.2 [4.9–202.1] | 27 (19.1%) | 61 (43.3%) | 76 (53.9%) |

| Table S3: Mortality Timing Stratified by Admission SOFA Categories in Non-Transitioned Patients | | | | | | |
| --- | --- | --- | --- | --- | --- | --- |
| SOFA category | **N patients** | **N deaths** | **Median death time [IQR], days** | **Deaths <5d** | **Deaths ≤30d** | **Deaths ≤90d** |
| ≤8 | 33 | 21 | 44.4 [19.5–357.4] | 2 (6.1%) | 7 (21.2%) | 13 (39.4%) |
| 9–11 | 44 | 32 | 19.6 [6.3–465.4] | 8 (18.2%) | 19 (43.2%) | 22 (50.0%) |
| 12–14 | 30 | 25 | 16.1 [5.3–48.0] | 6 (20.0%) | 16 (53.3%) | 20 (66.7%) |
| ≥15 | 34 | 27 | 12.1 [2.5–46.8] | 11 (32.4%) | 19 (55.9%) | 21 (61.8%) |

# Discussion

The finding that metabolic transition is associated with improved 90-day survival supports the concept that resolution of the catabolic state marks a turning point in critical illness. Transition represents not only improved insulin sensitivity but also parallel recovery in hemodynamic stability, inflammation, and organ function. The consistency of survival benefit across different landmark times strengthens confidence that this is a robust phenomenon rather than a statistical artifact.

Transition from catabolic to anabolic state was associated with improved survival regardless of admission SOFA or baseline characteristics. The never-transitioned group carried the highest mortality risk, a pattern not fully explained by baseline SOFA or the frequency of mechanical ventilation. Instead, their poor prognosis was driven by a dual phenomenon: very early deaths that precluded transition, and persistent failure to recover despite surviving beyond the early phase. These findings highlight that admission severity scores alone cannot capture metabolic recovery potential, and underscore the importance of transition detection as a prognostic marker.

**Limitations** should be acknowledged: this is a retrospective analysis, transition status was algorithmically defined and may be sensitive to glucose sampling frequency or data completeness, and residual confounding cannot be excluded.

Nevertheless, the observed above 40% reduction of the mortality risk suggests that metabolic transition could be a clinically meaningful surrogate of recovery and a potential target for prospective interventional studies. Failure to achieve transition was associated with substantially worse 90-day survival, underscoring its prognostic importance.
