# Supplementary material for: Detecting the metabolic transition to personalize nutritional timing: model development and preliminary validation in a large ICU cohort
Source: Crit Care. 2026 Feb 24;30:132. doi: 10.1186/s13054-026-05874-5 (PMC13037178; doi:10.1186/s13054-026-05874-5)
Supplement: Supplementary file 4 — Supplementary Material 4 [file 13054_2026_5874_MOESM4_ESM.docx]

Supplement 4- Mortality effect of overfeeding before transition

# **Methods**

## **Nutrition exposure definition**

- **High nutrition exposure:** patients with nutrition ≥1 kcal/kg/h for at least 24 hours prior to metabolic transition.
- **Low nutrition exposure:** patients who remained <1 kcal/kg/h during the entire pre-transition period.
- **Intermediate exposure:** patients with some hours ≥1 kcal/kg/h but <24 hours; these were excluded from primary analyses unless specified.

## **Transition definition**

- Transition was defined as the timepoint determined by the insulin resistance index (IRI) drop model.
- Analyses were anchored on transition day.

## **Covariates**

- **Age:** continuous, years.
- **Gender:** mapped as male vs female.
- **SOFA score:** total score at admission.
- **Admission diagnoses:** individual diagnosis columns (sepsis, septic shock, pneumonia, cardiogenic shock, multi-trauma, etc.).

## **Mortality outcome**

- **Primary endpoint:** all-cause mortality at 90 days after ICU admission.
- Death time was recorded in days from admission, censored at 90 days.

## **Statistical analysis**

- Logistic regression for 90-day mortality with odds ratios (OR) and 95% confidence intervals (CI).
- Adjusted covariates: age, gender, SOFA score, admission diagnoses- sepsis, septic shock, cardiogenic shock, pneumonia, Trauma-MVA.
- Model diagnostics: complete case analysis.
- Sensitivity analyses varied the kcal/kg/h cutoff (0.6, 0.8,1.0, 1.2, 1.4) thresholds.

# **Supplementary Tables**

| Threshold (kcal/kg/h) | n High | Mortality High % | n Low | Mortality Low % | Difference (High–Low) % |
| --- | --- | --- | --- | --- | --- |
| 0.6 | **936** | **42.9%** | **717** | **34.4%** | **+8.5** |
| 0.8 | **830** | **43.0%** | **871** | **35.6%** | **+7.4** |
| 1.0 | **644** | **44.7%** | **1,096** | **36.5%** | **+8.2** |
| 1.2 | **412** | **47.6%** | **1,329** | **36.9%** | **+10.6** |
| 1.4 | **227** | **51.5%** | **1,549** | **37.4%** | **+14.2** |

# **Supplementary Figures**


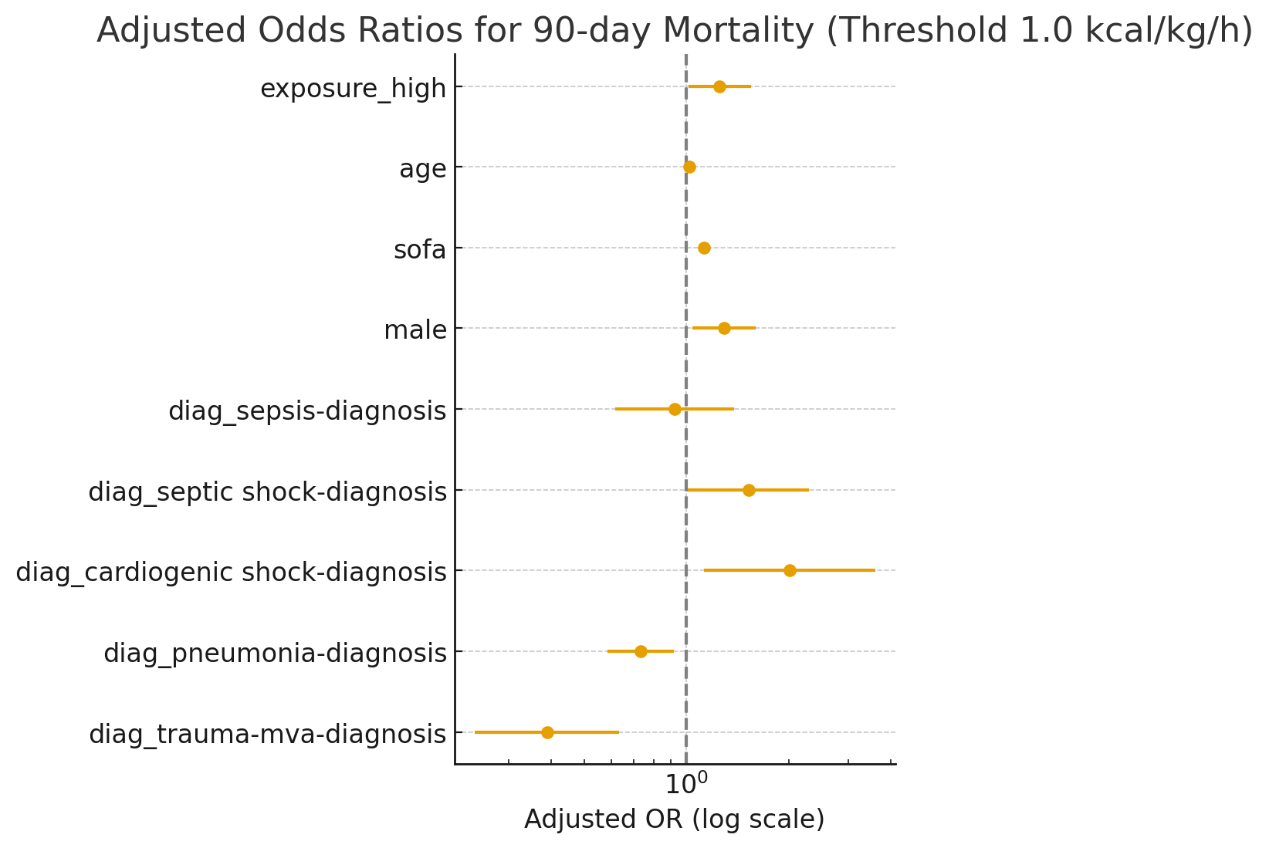


**Figure S1- Adjusted odds ratios for 90-day mortality at the 1.0 kcal/kg/h threshold.**
Forest plot of the multivariable logistic regression model including age, gender, SOFA score, and admission diagnoses. The primary exposure, **high pre-transition nutrition (≥1.0 kcal/kg/h for ≥24 h before transition)**, was associated with increased 90-day mortality (OR 1.25, 95% CI 1.01–1.55, p=0.038). Odds ratios (dots) are shown with 95% confidence intervals (horizontal bars) on a logarithmic scale; the dashed vertical line denotes the null value (OR=1).


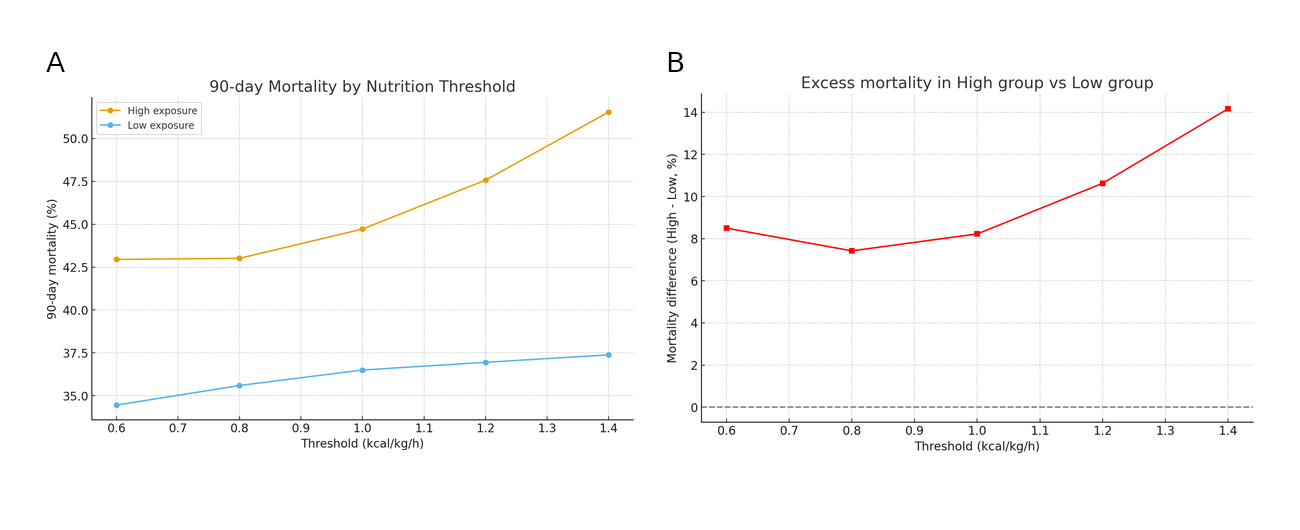


**Figure S2 — Mortality by Thresholds**

**Figure S2A. Ninety-day mortality by exposure group across thresholds (0.6–1.4 kcal/kg/h).**
Mortality rates in the **High nutrition group (≥ threshold for ≥24 h)** and **Low group (< threshold)** are plotted for each cutoff. Mortality in the High group consistently exceeded that of the Low group, with divergence increasing at higher thresholds.

**Figure S2B. Excess mortality (High–Low) across thresholds.**
Absolute mortality difference between High and Low groups, expressed as percentage points, plotted against the kcal/kg/h threshold. The mortality gap widened progressively from ~7–9% at 0.6–1.0 kcal/kg/h to ~14% at ≥1.4 kcal/kg/h, suggesting a dose–response relationship.

# **Supplementary Discussion**

The adjusted analyses consistently demonstrated that **high pre-transition nutrition exposure was associated with increased 90-day mortality**, with the strength of association becoming more pronounced at higher caloric thresholds. At the conventional cutoff of **1.0 kcal/kg/h**, high exposure was linked to a **25% increase in the odds of death** (OR 1.25, 95% CI 1.01–1.55, p=0.038). Sensitivity analyses across thresholds of **0.6, 0.8, 1.0, 1.2, and 1.4 kcal/kg/h** showed a consistent excess risk in the high nutrition group, with absolute mortality differences ranging from **~7–9% at the lower cutoffs** to **14% at ≥1.4 kcal/kg/h**, suggesting a dose–response relationship.

Exploratory **piecewise Cox models** indicated that this association was **time dependent**: during the first 30 days, high pre-transition nutrition was paradoxically associated with **lower hazard of death** (HR 0.75, 95% CI 0.62–0.90), but between 30–90 days it was associated with a **marked increase in mortality risk** (HR 1.78, 95% CI 1.34–2.37). This dynamic effect explains the crossing of Kaplan–Meier curves and highlights that the apparent early benefit is outweighed by subsequent late harm.

Together, these findings support the hypothesis that the pre-transition metabolic state is not suited for full caloric provision and that overfeeding before transition may be harmful. Limitations include the retrospective design, potential residual confounding, and reliance on accurate coding of nutrition exposure and diagnoses. Nonetheless, the consistency of results across multiple thresholds, analytic approaches, and time intervals strengthens confidence in the validity of the findings.
