# Supplementary material for: Detecting the metabolic transition to personalize nutritional timing: model development and preliminary validation in a large ICU cohort
Source: Crit Care. 2026 Feb 24;30:132. doi: 10.1186/s13054-026-05874-5 (PMC13037178; doi:10.1186/s13054-026-05874-5)
Supplement: Supplementary file 5 — Supplementary Material 5 [file 13054_2026_5874_MOESM5_ESM.docx]

# Supplement 5: Feature Importance Analysis

This supplement details the feature importance analysis conducted on the model configured with a 30% IRI drop threshold, requirement of at least 2 out of 8 criteria, and without inclusion of nutrition in the formula. Feature importance here refers to the relative contribution of each physiological marker to the identification of the catabolic-to-anabolic transition.

## 1. Frequency of Criteria Contribution

The first analysis quantified how frequently each marker contributed to confirming a transition across all included patients. Albumin and lactate were the most frequently used criteria, followed by noradrenaline, CRP, and WBC.

| **Criterion** | **Count** | **Percentage (%)** |
| --- | --- | --- |
| Albumin | 2122 | 90.3 |
| Lactate | 1846 | 78.6 |
| Noradrenaline | 1566 | 66.6 |
| CRP | 1177 | 50.1 |
| WBC | 1091 | 46.4 |
| Vasopressin | 747 | 31.8 |
| Adrenaline | 262 | 11.1 |
| Neutrophils% | 220 | 9.4 |

## 2. Sensitivity Analysis (Marker Removal)

To assess the relative impact of each marker on transition detection, a sensitivity analysis was performed. Each marker was removed individually, and the model was re-applied to determine how many patients still fulfilled the transition criteria. Markers whose removal led to greater losses in detected transitions are considered more influential. Albumin and lactate again showed the strongest influence.

| Criterion | Patients Still Transitioned | Patients Lost Transition | Loss (%) |
| --- | --- | --- | --- |
| Albumin | 1972 | 378 | 16.1 |
| Lactate | 2041 | 309 | 13.1 |
| CRP | 2162 | 188 | 8.0 |
| Noradrenaline | 2169 | 181 | 7.7 |
| WBC | 2175 | 175 | 7.4 |
| Vasopressin | 2197 | 153 | 6.5 |
| Adrenaline | 2205 | 145 | 6.2 |
| Neutrophils% | 2207 | 143 | 6.1 |

## Conclusions

Feature importance analysis demonstrated that albumin and lactate were the most critical markers for confirming transition, with their removal leading to losses in 16% and 13% of patients respectively. CRP, noradrenaline, and WBC had a moderate contribution, while adrenaline, vasopressin, and neutrophils were less decisive in this iteration. These findings highlight that inflammatory and hemodynamic stabilization — in particular, the absence of further albumin decline (as an inflammatory stress marker) together with lactate clearance — are central signals driving the model’s identification of transition
